# Supplementary material for: Transferable Implicit Solvation via Contrastive Learning of Graph Neural Networks
Source: ACS Cent Sci. 2023 Nov 16;9(12):2286–97. doi: 10.1021/acscentsci.3c01160 (PMC10755853; doi:10.1021/acscentsci.3c01160)
Supplement: Supplementary file 1 — oc3c01160_si_001.pdf [file oc3c01160_si_001.pdf]

Supporting Information: Transferable Implicit Solvation via Contrastive Learning of Graph Neural Networks

Justin Airas[1], Xinqiang Ding[1], and Bin Zhang[1,*]

[1] *Department of Chemistry, Massachusetts Institute of Technology, Cambridge, MA, USA*

* Email: binz@mit.edu

Contents

Selecting data samples from explicit solvent MD simulations **S-1**

Umbrella sampling simulation set-up **S-2**

Umbrella simulations with GBn2 to generate noise configurations S-3

MD simulations for proteins outside of the training set S-5

ML-MD simulations using the SchNet implicit solvent S-6

Benchmarking simulations S-6

Software versions & hardware S-7

Pre-training & potential contrasting set-up **S-8**

Loss functions S-9

Hyperparameters S-10

Software versions & hardware S-12

Noise potential & free energy profile calculations **S-13**

Constructing the noise potential using FastMBAR S-13

Free energy profile calculations S-14

Calculating free energy profiles from umbrella simulations using FastMBAR . S-14

Calculating free energy profiles from unbiased explicit solvent simulations . . S-15

TICA free energy profiles S-16

Possible causes of lower accuracy for BBA, WW domain, & NTL9 **S-17**

Figures **S-19**

Table S1: Interval, RMSD cutoff, and numbers of removed configurations for each protein for the selection of data samples. The RMSD removed row indicates the number of frames removed based on the selected RMSD cutoff, while the front and end removed rows indicate the number of configurations discarded from the beginning and the end of each ensemble. This selection process resulted in 100,000 data configurations for each protein. See text section *Selecting data samples from explicit solvent MD simulations* for further details.

<table>
<tr><th>System</th><th>CLN025</th><th>Trp-cage</th><th>BBA</th><th>Villin</th><th>WW domain</th><th>NTL9</th></tr>
<tr><td>Interval[a]</td><td>5</td><td>5, 2</td><td>8, 2</td><td>5</td><td>25, 2</td><td>67, 2</td></tr>
<tr><td>RMSD cutoff (nm)[b]</td><td>1</td><td>1.5</td><td>1.75</td><td colspan="3">2</td></tr>
<tr><td>RMSD removed</td><td colspan="4">0</td><td>88</td><td>4</td></tr>
<tr><td>Front removed</td><td>3,000</td><td>4,400</td><td>1,250</td><td>12,000</td><td>13,550</td><td>10,500</td></tr>
<tr><td>End removed</td><td>3,000</td><td>4,400</td><td>1,250</td><td>12,000</td><td>13,562</td><td>10,596</td></tr>
</table>

[a] If listed, a 2nd interval was used to select frames after RMSD-based, front, and end configuration removal.

[b] RMSD cutoff selected based on RMSD ranges from umbrella sampling MD (Table S2).

Selecting data samples from explicit solvent MD simulations

Atomistic TIP3P[S1] explicit solvent molecular dynamics (MD) simulations for chignolin CLN025 (CLN025), Trp-cage, BBA, Villin, WW domain, and NTL9 were conducted by Lindorff-Larsen et al.[S2] From each of the six ensembles, we selected 100,000 configurations as data samples for training. The process for selecting data samples for each protein was as follows: first configurations were selected at a specific interval, next a small number of configurations beyond a specific peptide backbone heavy atom root-mean-squared-deviation (RMSD) distance cutoff from the folded structure $\boldsymbol{x}_\circ$ were removed, and lastly a number of configurations were removed from the beginning and the end of the ensembles to ensure exactly 100,000 configurations for all proteins. Configurations beyond the RMSD cutoff were removed as these fell outside of the range covered by the noise configurations. Few configurations fell beyond RMSD the cutoff, if at all. All selection intervals, RMSD cutoffs, and beginning (front)- and end-of-ensemble removed frames are detailed in Table S1.

Due to the 200 ps save interval used by Lindorff-Larsen et al.[S2], our selection procedure

results in the following time intervals between each data configuration: 1 ns for CLN025 and Villin, 2 ns for Trp-cage, 3.2 ns for BBA, 10 ns for WW domain, and 26.8 ns for NTL9.

k-means clustering (using the `cluster.kmeans` function of the pytraj package[S3]) was used to identify $\boldsymbol{x}_\circ$ for each protein. The centroid frame of the most populated of $k = 10$ computed clusters was selected as $\boldsymbol{x}_\circ$ for each protein. A similar process was used to identify $\boldsymbol{x}_\circ$ in the original study by Lindorff-Larsen et al.[S2]. The structures we identified as $\boldsymbol{x}_\circ$ match those identified by Lindorff-Larsen et al.[S2]

Umbrella sampling simulation set-up

RMSD-biased umbrella sampling MD simulations were conducted for each protein in the training set using the GBn2 implicit solvent model model.[S4] We also performed similar simulations using the trained SchNet implicit solvent model.

The RMSD was computed in reference to the folded structures, $\boldsymbol{x}_\circ$, identified using a procedure detailed in the previous section *Selecting data samples from explicit solvent MD simulations*. Only peptide backbone heavy atoms were used to compute the RMSD. A harmonic RMSD-biasing potential

$$u^i_{\text{bias}}(\boldsymbol{x}) = \frac{1}{2} k^i_{\text{RMSD}} \left[\text{RMSD}(\boldsymbol{x}, \boldsymbol{x}_\circ) - \text{RMSD}^\circ_i\right]^2 \tag{S1}$$

was applied to the ith umbrella window. A total of M windows were sampled for each protein. The RMSD centers, RMSD°_i, for each protein were evenly spaced at an interval of 0.05 nm within the RMSD range indicated in the tables that follow. RMSD ranges were selected to cover the full configurational space of each protein (Figure S15).

For all umbrella sampling simulations (with exceptions detailed later), a force constant $k^i_{\text{RMSD}} = 15,000$ kJ/mol/nm^2 was applied to the first two ($i = 1$ and $i = 2$) and the last two ($i = M - 1$ and $i = M$) windows within the indicated RMSD ranges. $k^i_{\text{RMSD}} = 12,500$ kJ/mol/nm^2 was applied to the next two ($i = 3$ and $i = 4$) and the next-to-last two ($i = M-3$

Table S2: Parameters for RMSD-biased umbrella sampling MD simulations for proteins within the training set.

System	CLN025	Trp-cage	BBA	Villin	WW domain	NTL9
Solv. model	GB-neck2 (GBn2)[S4]					
Force fields[a]	CHARMM36 (C36)[S6]			C36m[S7]<br>C36[S8]	C36[S6]	C36m[S7]<br>C36[S8]
Integrator	Langevin Middle[S9]					
Temperature (k)[b]	340	290	325	360		355
Friction γ (ps^{-1})	1					
Constraints	H bonds[c]					
H-mass (amu)	1					
Nonbond scheme	No cutoff					
Time step (fs)	2.5					
Minimize steps	50,000					
Equilibrate steps	1,200,000					
Traj. length (ns)[d]	1,060	1,392	225	155	284	276.25
Save interval (ps)	200		40	50		
RMSD (nm)	0 - 0.95	0 - 1.45	0 - 1.75	0 - 1.95		
M[e]	20	30	36	40		

[a] CHARMM36m[S7] (C36m) and C36 for nonstandard amino acids[S8] were used to parameterize norleucine residues in villin and NTL9. C36 for nonstandard amino acids was validated for use with C36m.[S8]
[b] Same temperatures as Lindorff-Larsen et al.[S2] simulations.
[c] H-X bond lengths constrained.
[d] Length of each umbrella window trajectory (traj.).
[e] Total # of umbrella windows.

and $i = M - 2$) windows within the indicated ranges. For all other windows, $k^i_{\mathrm{RMSD}} = 10,000$ kJ/mol/nm^2 was applied.

Minimization and equilibration were performed prior to all production simulations. Minimization was performed using the `minimizeEnergy` method of the `Simulation` class in OpenMM.[S5] Equilibration was performed by iteratively increasing k^i_{RMSD} to its production value. For each protein, the tables that follow detail the total number of minimization and equilibration steps and time steps, respectively.

Table S3: Selection ranges and intervals of noise sample configurations. The process for selecting noise sample configurations was as follows: first configurations were removed from the beginning of each umbrella window, next the remaining configurations were concatenated into a single umbrella ensemble, and lastly configurations were selected from the umbrella ensemble at a specific interval. Ranges of selected configurations are shown for each umbrella window, while the interval at which frames were selected are shown for the entire umbrella ensemble. An additional frame was occasionally removed from some windows to ensure 100,000 configurations for each protein.

**System**	**CLN025**	**Trp-cage**	**BBA**	**Villin**	**WW domain**	**NTL9**
Range(s)	[300:]	[293:], [294:][a]	[69:], [70:][b]	[600:]	[680:]	[525:]
Interval	1	2		1	2	

[a] An additional frame was removed from windows in range 1 - 1.45 nm.
[b] An additional frame was removed from windows in range 1 - 1.75 nm.

Umbrella simulations with GBn2 to generate noise configurations

RMSD-biased umbrella sampling MD simulations were conducted to generate noise samples for the following training set proteins: CLN025, Trp-cage, BBA, Villin, WW domain, and NTL9. Table S2 details the simulation parameters, number of minimization steps, number of equilibration time steps, RMSD ranges, and total number of umbrella windows M used for these umbrella sampling simulations. For each protein, the interval and range of noise sample configurations selected from each umbrella ensemble are detailed in Table S3. From the resultant umbrella ensembles, 100,000 configurations for each protein were used as noise samples for training with the potential contrasting[S10] method.

Note that for CLN025 and Trp-cage, each umbrella trajectory is of significantly longer timescale than for the remaining proteins in the training set (Table S2). While it was initially unknown what timescale would be necessary to achieve convergence of the free energy profile with respect to RMSD for each protein, later analysis revealed that such long timescales were unnecessary. As such, trajectories for the remaining proteins are of shorter timescale and save configurations at a higher frequency. Figure S15 suggests that our noise configurations sufficiently sample the RMSD phase space covered by the data configurations. Free energy profile convergence for each protein is demonstrated in Figure S16, while the process for

Table S4: Parameters used for TIP3P and GBn2 RMSD-biased umbrella sampling MD simulations for proteins outside of the training set.

**System**	**1uao**		**T8P**		**JIP1 10-mer**
Solv. model	TIP3P[S1]	GBn2[S4]	TIP3P[S1]	GBn2[S4]	GBn2[S4]
Force fields	C36[S6]				ff14SB[S11]
Integrator	Langevin Middle[S9]				
Temperature (K)	315[a]		420[b]		300[c]
Friction γ (ps^{-1})	1				0.01
Constraints	H bonds[d]				All bonds
H-mass (amu)	1				4
Nonbond scheme	PME	No cutoff	PME	No cutoff	No cutoff
Nonbond cutoff (nm)	1.2	-	1.2	-	-
Switch dist. (nm)	1	-	1	-	-
Solv. padding (nm)	2.4	-	2.4	-	-
Time step (fs)	2.5				5
Minimize steps	50,000				
Equilibrate steps	1,200,000				1,100,000
Traj. length (ns)[e]	62.5				1,000
Save interval (ps)	25				20
RMSD (nm)	0 - 0.95				
M^f	20				

[a] Denaturation midpoint (melting temperature) for 1uao.
[b] Gly7-Pro8 *cis* to *trans* transition temperature for T8P 1uao mutant.
[c] Same temperature used by Ojaghlou et al.[S12]
[d] H-X bond lengths constrained.
[e] Length of each umbrella window traj.
[f] Total # of umbrella windows.

computing these free energy profiles is detailed in section *Noise potential & free energy profile calculations*.

MD simulations for proteins outside of the training set

Table S4 details the simulation parameters, number of minimization steps, number of equilibration time steps, RMSD ranges, and total number of umbrella windows M for umbrella sampling simulations used to generate atomistic, TIP3P explicit solvent and GBn2 implicit solvent configurations for the following proteins: chignolin 1uao (PDB ID: 1uao),[S13] a T8P mutant of chignolin 1uao that does not favor the native hairpin structure,[S14] and a

structurally-active 10-mer of the C-Jun amino-terminal kinase-interacting protein 1 (JIP1).[S12] These proteins were used to evaluate the transferability of the SchNet implicit solvent model outside the training set. The explicit solvent configurations were generated by Ojaghlou et al.[S12] for the JIP1 10-mer and by new umbrella sampling MD simulations for 1uao and the T8P mutant. GBn2 configurations were also generated using umbrella sampling MD simulations for all three proteins. Since there are 18 individual structures associated with the PDB ID 1uao, the structure with the lowest gas-phase energy (the 15th structure) was used as $\boldsymbol{x}_\circ$. The same structure (albeit with the T8P mutation) was used as $\boldsymbol{x}_\circ$ for the T8P mutant. The T8P mutant was generated by using the `PDB Reader & Manipulator` tool in CHARMM-GUI[S15–S17] to mutate the selected 1uao PDB structure. While the JIP1 10-mer lacks a distinct folded state, the compact structure of Conformer 1 as identified by Ojaghlou et al.[S12] was used as $\boldsymbol{x}_\circ$. Free energy profile convergence for umbrella sampling simulations of these proteins is displayed in Figure S17.

ML-MD simulations using the SchNet implicit solvent

Table S5 details the simulation parameters, number of minimization steps, number of equilibration time steps, RMSD ranges, and total number of umbrella windows M used for RMSD-biased umbrella sampling simulations using the SchNet implicit solvent. Umbrella sampling was conducted to enable the calculation of free energy profiles with respect to RMSD from $\boldsymbol{x}_\circ$ for comparison to the results from atomistic TIP3P and GBn2 simulations. Note that while our SchNet implicit solvent model was developed with the intention of using it with C36, ff14SB[S11] was used for the JIP1 10-mer since the explicit solvent configurations were generated by Ojaghlou et al.[S12] using ff14SB. Free energy profile convergence for these simulations is displayed in Figure S18.

Table S5: Parameters used for RMSD-biased umbrella sampling ML-MD simulations using the SchNet implicit solvent. Parameters for CLN025, Trp-cage, 1uao chignolin, T8P 1uao chignolin, and the JIP1 10-mer are shown.

System	CLN025	Trp-cage	1uao	T8P	JIP1 10-mer
Solv. model	SchNet implicit solvent				
Force fields	C36[S6]				ff14SB[S11]
Integrator	Langevin Middle[S9]				
Temperature (K)	340	290	315	420	300
Friction γ (ps^{-1})	1		1		0.01
Constraints	All bonds		All bonds		
H-mass (amu)	4				
Nonbond scheme	No cutoff				
Time step (fs)	5				
Minimize steps	5,000				1,000
Equilibrate steps	540,000	600,000	540,000		
Traj. length (ns)^a	13	20	13		30
Save interval (ps)	25	20	25		10
RMSD (nm)	0 - 0.95	0 - 1.45	0 - 0.95		
M^b	20	30	20		

^{a} Length of each umbrella window traj.
^{b} Total # of umbrella windows.

Benchmarking simulations

Table S6 details the parameters of simulations used to evaluate the simulation speed of the SchNet implicit solvent. All simulations were run with a harmonic RMSD biasing potential (Eq. S1) centered at 0.0 nm from the folded structures. Compared with more extended conformations, folded structures produce more nearest neighbors per atom when defining the graph, creating a worst-case scenario for the SchNet implicit solvent model.

Software versions & hardware

MD simulations with TIP3P explicit solvent or GBn2 implicit solvent were conducted using OpenMM[S5] version (ver.) 7.7.0 on a single Nvidia Volta V100 GPU. ML-MD simulations with the SchNet implicit solvent model were conducted using OpenMM[S5] ver. 8.0.0 beta and OpenMM PyTorch plugin[S18] ver. 1.0 beta with PyTorch[S19] ver. 1.11.0 and PyTorch

Table S6: Parameters for MD and ML-MD simulations used for benchmarking.

**System**	**CLN025**		**Trp-cage**		**BBA**	
Solv. model	Vacuum / GBn2[S4]/ SchNet	TIP3P[S1]	Vacuum / GBn2[S4]/ SchNet	TIP3P[S1]	Vacuum / GBn2[S4]/ SchNet	TIP3P[S1]
Force fields	ff14SB[S11]					
Integrator	Langevin Middle[S9]					
Temperature (K)	340		290		325	
Friction γ (ps^{-1})	1		1		0.01	
Constraints	All bonds		All bonds		H Angles[a]	
H-mass (amu)	4					
Nonbond scheme	No cutoff	PME	No cutoff	PME	No cutoff	PME
Nonbond cutoff (nm)	-	1.2	-	1.2	-	1.2
Switch dist. (nm)	-	1	-	1	-	1
Solv. padding (nm)	-	2.4	-	2.4	-	2.4
Time step (fs)	5					
Minimize steps	5,000					
Equilibrate steps	540,000		600,000		250,000	
Traj. length (ns)	12		7		0.875	
Save interval (ps)	25		20		0.5	
RMSD center (nm)	0.0		0.0		0.0	

[a] All bond lengths, and all H-X-H and H-O-X angles fixed

Cluster[S20] ver. 1.5.9. Due to software dependency limitations, ML-MD simulations were run on Intel Xeon CPUs, with 4 cores for CLN025, 12 cores for Trp-cage, 12 cores for 1uao, 8 cores for T8P 1uao, and 4 cores for the JIP1 10-mer. Trajecotry analyses such as RMSD and *k*-means clustering were performed using both pytraj[S3] ver. 2.0.6 and MDtraj[S21] ver. 1.9.7.

Pre-training & potential contrasting set-up

Loss functions

The root-mean-squared-error (RMSE) loss function was used for pre-training the SchNet[S22] model to fit E_{GBn2}. This was implemented in PyTorch[S19] as the square root of the `MSELoss` class

$$\mathrm{RMSE} = \sqrt{\frac{\sum_{i=1}^{N_{\mathrm{set}}} \left[E^{i}_{\mathrm{GBn2}}\left(\boldsymbol{x}\right) - E^{i}_{\mathrm{SchNet}}\left(\boldsymbol{x};\boldsymbol{\theta}\right)\right]^2}{N_{set}}}, \tag{S2}$$

where N_{set} is the total number of configurations in the training / testing sets. The dataset used to pre-train SchNet was composed of 100,000 atomistic configurations and 100,000 GBn2 configurations per protein. These were the same configurations used to train SchNet with the potential contrasting method. The testing set held aside when pre-training SchNet was composed of 50,000 configurations. As such, for the training set $N_{\mathrm{set}} = 150,000$ and for the testing set $N_{\mathrm{set}} = 50,000$.

The objective function used in potential contrasting is defined as

$$\begin{aligned}\ell_{\mathrm{tot}}\left(\boldsymbol{\theta}, \{\boldsymbol{\Delta F}\}_{k=1}^{K}\right) = \sum_{k=1}^{K} \frac{1}{N_p^k} \Bigg\{ \sum_{i=1}^{N_p^k} \ln\left[\frac{1}{1+\nu_k \exp\left(-\beta\left[u_q^k\left(\boldsymbol{x}_p^{ki}\right) - u_p\left(\boldsymbol{x}_p^{ki};\boldsymbol{\theta}\right) + \boldsymbol{\Delta F}_k\right]\right)}\right] + \\ \sum_{i=1}^{N_q^k} \ln\left[\frac{1}{1+\nu_k^{-1} \exp\left(-\beta\left[u_p\left(\boldsymbol{x}_q^{ki};\boldsymbol{\theta}\right) - u_q^k\left(\boldsymbol{x}_q^{ki}\right) - \boldsymbol{\Delta F}_k\right]\right)}\right]\Bigg\},\end{aligned} \tag{S3}$$

where k indices over proteins in the training set. $\boldsymbol{\Delta F}_k = \boldsymbol{F}_p^k - \boldsymbol{F}_q^k$ and $\nu_k = \frac{P(y=0)}{P(y=1)} = \frac{N_q}{N_p}$. $\boldsymbol{\Delta F}$ is a free parameter during training. When training SchNet with potential contrasting, the bias parameter of the final layer of the feed-forward NN is held constant to prevent it from counter-acting $\boldsymbol{\Delta F}$. $u_q^k\left(x\right)$ is the potential energy function corresponding with the noise distribution of the kth protein, while $u_p\left(\boldsymbol{x};\boldsymbol{\theta}\right) = U_{\mathrm{C36}}\left(x\right) + E_{\mathrm{SchNet}}\left(\boldsymbol{x};\boldsymbol{\theta}\right)$ is the potential energy corresponding with the learned CG distribution that approximates the atomistic distribution. U_{C36} is simply the potential energy computed from the CHARMM force field.[S6,S7]

We used the same number of data samples $N_p^k = 100{,}000$ and noise samples $N_q^k = 100{,}000$. As such $\nu_k = 1$ for all proteins in our training set and will be omitted from all equations that follow. Equation S3 was implemented in PyTorch as the sum of individual binary cross entropy (BCE) with log-odds-ratios (logits) loss functions for each protein

$$\mathrm{BCE}\left(\boldsymbol{\theta}, \{\boldsymbol{\Delta F}\}_{k=1}^{K}\right) = \sum_{k=1}^{K} \left\{ \frac{1}{N_{tot}^k} \sum_{i=1}^{N_{tot}^k} y_{ki} \ln\left[\sigma\left(\mathrm{logit}\left(\boldsymbol{x}^{ki}\right)\right)\right] + (1 - y_{ki}) \ln\left[1 - \sigma\left(\mathrm{logit}\left(\boldsymbol{x}^{ki}\right)\right)\right] \right\}, \quad \text{(S4)}$$

where $\sigma(z) = 1/\left[1 + \exp(-z)\right]$ is the sigmoid function and y is a label that indicates whether $\boldsymbol{x}^{ki}$ is a data sample ($y = 1$) or noise sample ($y = 0$). $N_{\mathrm{tot}}^k = N_p^k + N_q^k$ was the total number of configurations for the kth protein. Each individual BCE with logits loss term was implemented with the `BCEWithLogitsLoss` class in PyTorch. By inserting the logit of $\boldsymbol{x}_{ki}$ for our learned $p(\boldsymbol{x}; \boldsymbol{\theta})$ and our noise $q_k(\boldsymbol{x})$ distributions,

$$\mathrm{logit}\left(\boldsymbol{x}^{ki}\right) = \ln\left(\frac{p\left(\boldsymbol{x}^{ki}; \boldsymbol{\theta}\right)}{q_k\left(\boldsymbol{x}^{ki}\right)}\right) = \ln\left(\frac{\exp\left(-\beta\left[u_p\left(\boldsymbol{x}^{ki}; \boldsymbol{\theta}\right) - \boldsymbol{F}_p^k\right]\right)}{\exp\left(-\beta\left[u_q^k\left(\boldsymbol{x}^{ki}\right) - \boldsymbol{F}_q^k\right]\right)}\right) = -\beta\left[u_p\left(\boldsymbol{x}^{ki}; \boldsymbol{\theta}\right) - u_q^k\left(\boldsymbol{x}^{ki}\right) - \boldsymbol{\Delta F}_k\right], \quad \text{(S5)}$$

into $\sigma(z)$, it is clear that equations S3 and S4 are effectively equivalent.

An L2 regularization term $\lambda \sum_{i=1}^{N_{\mathrm{parms}}} \boldsymbol{\theta}_i^2$ (where N_{parms} is the total number of trainable parameters in SchNet, $\boldsymbol{\theta}_i$ represents the ith trainable parameter, and λ represents the regularization constant hyperparameter) was added to both loss functions (equations S2 and S4) during training to help prevent overfitting. This was implemented in PyTorch by specifying a `weight_decay` value within the `optim.Adam` class.

Hyperparameters

Table S7 details all explored SchNet architectures,[S22] while Table S8 details the training set-up hyperparameters (learning rates ζ, L2 regularization constants λ, batch sizes, etc.). As discussed in the main text, the cutoff distance used for defining nearest neighbors (r_{cut}) and the number of interaction blocks (N_{IB}) are the most impactful hyperparameters for the SchNet architecture. Increasing the embedding width only appeared to increase memory usage, and as such no rigorous evaluation of this hyperparameter was performed.

With all other optimal hyperparameters selected (Table S7 - S8), several SchNet implicit solvents with different values of the L2 regularization constant λ were trained. While not found to affect re-weighted free energy profiles, λ had a significant effect on the performance of the SchNet implicit solvent model in ML-MD simulations. Unlike when performing re-weighting, computing free energy profiles from ML-MD simulations involves entirely new configurations (i.e., not included in the training set). Since it is well-known that regularization can improve the ability of ML models to generalize to samples outside of the training set, this outsized role of λ is not unexpected.

For models that we attempted to run ML-MD simulations with, the optimal value of λ was selected by running umbrella simulations for CLN025 and computing free energy profiles with respect to RMSD from $\boldsymbol{x}_\circ$. For the hyperparameter combination used to generate the ML-MD simulation data discussed in the main text ($N_{\text{IB}} = 3$, $r_{\text{cut}} = 1.8$ nm), CLN025 free energy profiles computed from ML-MD for models with different values of λ are shown in Figure S19. The most accurate model ($\lambda = 1 \times 10^{-6}$) was used for all simulations discussed in the main text. For a SchNet implicit solvent model trained with the same hyperparameters but without holding the interaction block parameters constant, free energy profiles are shown in Figure S20. Lastly, for a smaller SchNet model ($N_{\text{IB}} = 2$, $r_{\text{cut}} = 1.8$ nm), free energy profiles are shown in Figure S21. While one of these models was accurate for CLN025 ($\lambda = 1 \times 10^{-3}$), it was inaccurate and unstable for Trp-cage (Figure S22). While we also attempted simulations with larger models, we did not search for an optimal λ for these. Note

Table S7: Hyperparameters explored for SchNet.[S22] Bolded values indicate hyperparameters for the best performing model as determined from re-weighted free energy profiles (see Figure 5), while underlined values indicate the hyperparameters of the smaller model used in ML-MD simulation.

SchNet hyperparameters	
Embedding width	**<u>32</u>**, 40, 48, 64, 128
r_{cut} (nm)	1, 1.2, 1.4, 1.6, <u>1.8</u>, 2, 3, 4, **5**
N_{IB}	2, **<u>3</u>**, 4, 5, 6

Table S8: Hyperparameters explored for training set-up. Hyperparameters are indicated for both pre-training (i.e., training SchNet to fit E_{GBn2}) and potential contrasting. Bolded values indicate optimal hyperparameters. In cases where only a single value is listed, no further optimization was necessary beyond the initially selected value.

**Hyperparameter**	**Pre-training**	**Potential contrasting**
ζ	$\mathbf{1 \times 10^{-3}}$	$\mathbf{1 \times 10^{-4}}$
$\boldsymbol{\Delta F}_k$ ζ^a	-	**0.75**
# epochs	**120**	**30**
λ ($\times 10^{-7}$)	**50**	0.1, 1, **10**, 50, 100, 500, 1,000, 10,000
Batch size	10, **20**, 25, 50	**10**, 20, 25
Frozen parameters	**none**	none, **interaction blocks**
Testing set size^b	**50,000**	-
Activation	**shifted softplus**[S22]	
Optimizer	**Adam**[S23]	

^{a} Separate learning rate used for $\boldsymbol{\Delta F}_k$'s.
^{b} Total # of configurations held out of the training set for each protein. Different random sets of configurations were held out every epoch.

that we also did not search for an optimal λ when pre-training SchNet to fit E_{GBn2}, as we did not run ML-MD simulations with pre-trained models. We instead simply used our initially selected value.

Software versions & hardware

All models were trained using PyTorch[S19] ver. 1.10.2 and PyTorch Geometric[S20] ver. 2.0.4. This version of PyG contains PyTorch Cluster ver. 1.6.0 and PyTorch Scatter ver. 2.0.9. Both pre-training and potential contrasting were performed across two Nvidia Volta V100

GPUs using the `DistributedDataParallel` class. All configurations for three proteins were loaded onto each individual GPU.

Noise potential & free energy profile calculations

The multi-state Bennett acceptance ratio[S24] (MBAR) and the unbinned weighted histogram analysis method[S25] (UWHAM), as implemented in the FastMBAR package,[S26] were respectively used to compute the noise potential $u_q(\boldsymbol{x})$ as defined in the potential contrasting loss function (Eq. S3) and the free energy profile from the states sampled in biased umbrella sampling simulations. Free energy profiles were also computed from long timescale unbiased MD simulations. The methods used to compute $u_q(\boldsymbol{x})$ and these free energy profiles are detailed in the subsections below.

Constructing the noise potential using FastMBAR

As detailed in section *Umbrella sampling simulation set-up* and shown in equation S1, each window in RMSD-biased umbrella sampling simulations has a different biasing potential $u^i_{\text{bias}}(\boldsymbol{x})$ applied. The potential energy of the ith umbrella window in simulations with the GBn2 implicit solvent model is

$$u_i(\boldsymbol{x}) = U_{\text{ff}}(\boldsymbol{x}) + E_{\text{GBn2}}(\boldsymbol{x}) + u^i_{\text{bias}}(\boldsymbol{x}), \tag{S6}$$

where $U_{\text{ff}}(\boldsymbol{x})$ is the vacuum potential energy of the solute computed from the chosen protein force field and $E_{\text{GBn2}}(\boldsymbol{x})$ is the solvation free energy computed from GBn2.

Since a different potential energy function $u_i(\boldsymbol{x})$ was used for each umbrella window, a different distribution was sampled in each window. However, for training with potential contrasting,[S10] the noise samples must be drawn from a single noise distribution $q(\boldsymbol{x})$ yielded by a noise potential $u_q(\boldsymbol{x})$. As explained in the works of Ding et al.[S26] and Ding and Zhang[S10], by pooling all configurations from each umbrella window, they can treated as if they had

been drawn from a single generalized ensemble $p_{\mathrm{gm}}(\lambda = i, \boldsymbol{x}) \propto \exp\left(-\beta\left[u_i(\boldsymbol{x}) + v_i\right]\right)$. Here, λ is simply a discrete value representing the index of the ith state of $p_{\mathrm{gm}}(\lambda = i, \boldsymbol{x})$. The marginal probability density $p_{\mathrm{gm}}(\boldsymbol{x})$ on $\boldsymbol{x}$ in $p_{\mathrm{gm}}(\lambda = i, \boldsymbol{x})$ is yielded by the potential energy

$$u_{\mathrm{gm}}(\boldsymbol{x}) = -\beta^{-1} \ln \sum_{i=1}^{M} \exp\left(-\beta\left[u_i(\boldsymbol{x}) + v_i\right]\right), \tag{S7}$$

where v_i is a fitted biasing energy and M is the total number of states. Fitting of v_i is conducted so that the relative free energy G_i of the ith state of $p_{\mathrm{gm}}(\lambda = i, \boldsymbol{x})$ matches the relative population of configurations in the ith state of $p_{\mathrm{gm}}(\lambda = i, \boldsymbol{x})$. Thus, v_i must satisfy the condition $G_i = G_i^{*} + v_i = -\ln\left[n_i / N_{\mathrm{tot}}\right]$, where G_i^{*} is the relative free energy of the protein-solvent system in the ith thermodynamic state / umbrella window with the potential $u_i(\boldsymbol{x})$. These v_i's are fit by minimizing the convex objective function

$$f\left(v_1, v_2, ..., v_M\right) = \frac{1}{N_{\mathrm{tot}}} \sum_{j=1}^{M} \sum_{k=1}^{n_j} \ln\left[\sum_{l=1}^{M} \exp\left(-\beta\left[u_l\left(\boldsymbol{x}_j^k\right) + v_l\right]\right)\right] + \sum_{i=1}^{M} \frac{n_i}{N_{\mathrm{tot}}} v_i, \tag{S8}$$

where here $N_{\mathrm{tot}} = \sum_{i=1}^{M} n_i$. By solving for v_i we can compute $u_{\mathrm{gm}}(\boldsymbol{x})$ as shown in equation S7 and treat the marginal probability density $p_{\mathrm{gm}}(\boldsymbol{x})$ as our noise distribution $q(\boldsymbol{x})$. Thus, $u_{\mathrm{gm}}(\boldsymbol{x})$ is our noise potential energy $u_q(\boldsymbol{x})$.

In practice, fitting v_i and computing $u_q(\boldsymbol{x})$ was done using the FastMBAR package.[S26] This process of fitting v_i to compute $u_q(\boldsymbol{x})$ was done for each protein in our training set. As such, the kth protein has its own noise distribution $q_k(\boldsymbol{x})$ yielded by $u_q^k(\boldsymbol{x})$. These $u_q^k(\boldsymbol{x})$'s were used in the potential contrasting objective function (Eq. S3).

Free energy profile calculations

Free energy profiles were computed with respect to backbone heavy atom RMSD from the folded configuration $\boldsymbol{x}_{\circ}$ and with respect to the radius of gyration (Rg) of the backbone heavy atoms. The methods used to compute these free energy profiles are detailed below.

Calculating free energy profiles from umbrella simulations using FastMBAR

Computing v_i and $u_q(\boldsymbol{x})$ as detailed in section *Constructing the noise potential using FastMBAR* will enable usage of a simple re-weighting scheme to compute the free energy profile. While the following text details the computation of RMSD free energy profiles, the same procedure is used to compute Rg free energy profiles. The RMSD range can be split into L different bins, with RMSD_l representing the central RMSD of the lth bin. L was selected so that the width of the lth bin was 0.05 nm RMSD (or 0.0125 nm Rg). An energy of 0 or $+\infty$ kJ/mol is assigned to each configuration $\boldsymbol{x}$ according to the equation

$$R_l(\boldsymbol{x}) = \begin{cases} 0, & \text{if } \mathrm{RMSD}(\boldsymbol{x}, \boldsymbol{x}_\circ) \in [\mathrm{RMSD}_{l-0.5}, \mathrm{RMSD}_{l+0.5}] \\ +\infty, & \text{otherwise.} \end{cases}, \tag{S9}$$

where $\mathrm{RMSD}_{l-0.5}$ and $\mathrm{RMSD}_{l+0.5}$ are the lower and upper edges of the lth RMSD bin, respectively. After computing $R_l(\boldsymbol{x})$, we can compute the relative free energy of the lth bin

$$F_l = -\beta^{-1} \ln \left[\frac{1}{N_{\mathrm{tot}}} \sum_{j=1}^{M} \sum_{k=1}^{n_j} \exp\left(-\beta \left[R_l\left(\boldsymbol{x}_j^k\right) - u_q\left(\boldsymbol{x}_j^k\right) \right]\right) \right], \tag{S10}$$

where the summation runs over all configurations from all M umbrella windows.

For all free energy profiles computed in this way, the standard deviations were computed using block bootstrapping.[S27] Based on the save frequency of each simulation, the block size was selected as the number of consecutive configurations representing 2 ns of simulation. Ten bootstrap samples were constructed to compute each free energy profile.

Calculating free energy profiles from unbiased explicit solvent simulations

For protein configurations generated from long timescale, unbiased explicit solvent MD simulations, we computed free energy profiles directly from RMSD or Rg histograms with L total bins. L was selected so that the width of the lth bin was 0.05 nm RMSD or 0.0125 nm

Rg. This can be done by first computing the number of configurations n_l that fall within the RMSD or Rg range of the lth bin, next estimating the probability of configurations occurring within the lth bin as $p_l = n_l/N_{\text{tot}}$, and lastly by computing the relative free energy of the lth bin as $F_l = -\ln p_l$.

TICA free energy profiles

Following the procedure of Ojaghlou et al.[S12], dimensionality reduction was performed using time-lagged independent component analysis (TICA) on the explicit solvent configurations. Each backbone dihedral angle ω was featurized as pairs of $\sin(\omega)$ and $\cos(\omega)$, a lag time of 10 ns was selected, and the kinetic map option was set to false. The two outputted time-lagged independent components (TICs) were then binned to evenly spaced points on a grid matrix $L = (20 \times 20)$, and a kernel density estimate was computed over the grid points. At each individual grid point $\boldsymbol{X}_{i,j} = (x_i, y_j)$, the probability density

$$p_{i,j}\left(\boldsymbol{X}_{i,j}\right) = \frac{1}{N_c}\sum_{l=1}^{L_y}\sum_{k=1}^{L_x} c_{k,l} K_{\boldsymbol{H}}\left(\boldsymbol{X}_{i,j}, \boldsymbol{X}_{k,l}\right) \tag{S11}$$

was evaluated. Here, $c_{k,l}$ is simply the sum of the weights of each configuration assigned to the bin centered at $\boldsymbol{X}_{k,l}$, N_c is the sum of the weights of all configurations, and $K_{\boldsymbol{H}}\left(\boldsymbol{X}_{i,j}, \boldsymbol{X}_{k,l}\right)$ is a bivariate Gaussian kernel function with the bandwidth matrix $\boldsymbol{H}$ computed using Scott's Rule.[S28,S29] All explicit solvent configurations were uniformly weighted. The free energy for the bin centered at $\boldsymbol{X}_{i,j}$ was computed as $F_{i,j} = -\ln p_{i,j}$.

Free energy profiles were created for the GBn2 and SchNet implicit solvent umbrella sampling configurations by first fitting the backbone dihedrals from these configurations to the TICs computed for the explicit solvent data. Weights were then computed for each configuration. Following the procedure used to generate the noise potential $u_q(\boldsymbol{x})$ detailed in section *Constructing the noise potential using FastMBAR*, a generalized ensemble $p_{\text{gm}}(\lambda = i, \boldsymbol{x}) \propto \exp\left(-\beta\left[u_i(\boldsymbol{x}) + v_i\right]\right)$ was constructed and the energy of the corresponding marginal proba-

bility density $p_{\text{gm}}(\boldsymbol{x})$ on $\boldsymbol{x}$ in $p_{\text{gm}}(\lambda = i, \boldsymbol{x})$ was computed (Eq. S7). We then computed a weight $w(\boldsymbol{x})$ for each configuration. For configurations from GBn2 implicit solvent, $w(\boldsymbol{x}) = \frac{p(\boldsymbol{x})}{p_{\text{gm}}(\boldsymbol{x})} = \exp\left(-\beta\left[U_{\text{C36}}(\boldsymbol{x}) + E_{\text{GBn2}}(\boldsymbol{x}) - u_{\text{gm}}(\boldsymbol{x})\right]\right)$. For configurations from the SchNet implicit solvent, $w(\boldsymbol{x}) = \frac{p(\boldsymbol{x};\boldsymbol{\theta}^*)}{p_{\text{gm}}(\boldsymbol{x};\boldsymbol{\theta}^*)} = \exp\left(-\beta\left[U_{\text{C36}}(\boldsymbol{x}) + E_{\text{SchNet}}(\boldsymbol{x};\boldsymbol{\theta}^*) - u_{\text{gm}}(\boldsymbol{x};\boldsymbol{\theta}^*)\right]\right)$. Using these weights, free energy profiles were created by following the same procedure used to generate TICA free energy profiles for the JIP1 10-mer explicit solvent configurations.

Possible causes of lower accuracy for BBA, WW domain, & NTL9

As noted from Figure 5, the SchNet model trained using the potential contrasting method is less accurate for BBA, WW domain, and NTL9. This is likely caused by poor overlap of the noise distribution with the data distribution (for BBA) and by limitations of the SchNet architecture (for WW domain and NTL9).

While we initially evaluated noise distribution quality using RMSD histograms (Figure S15), structures with similar RMSD values from the folded state may still be highly divergent from each other. To further evaluate our noise distributions, we performed TICA dimensionality reduction on the pairwise distances between all Cα atoms for all training set proteins. We first mapped the data configurations to two TICs. The kinetic map option was set to false, and the lag time was set to 1 configuration (as detailed in section *Selecting data samples from explicit solvent MD simulations*, the time interval between data configurations ranges from 1 ns for CLN025 and Villin to 26.8 ns for NTL9). We then projected the Cα pairwise distances for our noise configurations to the two TICs. Plots of these TICs are displayed in Figure S23. Sufficient noise distribution overlap is noted for all proteins except BBA. This suggests that a low quality noise distribution may be the cause for lower accuracy of the transferable SchNet model for BBA (Figure 5). In future works aiming to develop more broadly-transferable ML CG force fields, noise distribution quality should be primarily

evaluated using TICA, and low quality noise should be replaced with more realistic noise generated using more rigorous enhanced sampling techniques.

To further investigate causes of decreased accuracy for BBA, WW domain, and NTL9, we trained three protein-specific SchNet models as implicit solvents using the potential contrasting method. We first pre-trained the three protein-specific models to fit E_{GBn2} for BBA, WW domain, and NTL9 configurations, respectively. The same hyperparameters ($r_{\mathrm{cut}} = 5$ nm, $N_{\mathrm{IB}} = 3$) used to train the transferable model were used for the protein-specific models. Free energy profiles for the protein-specific models are displayed in Figure S3. Significant improvement over the transferable model is noted for the NTL9-specific model. While some improvement is noted for the BBA-specific model, the previously discussed poor overlap between the BBA noise and data distributions likely prevents any more substantial improvement. Interestingly, while little improvement is noted for the WW domain-specific model, the free energy profile for this model closely aligns with GBn2 from 0 to $\sim$10 Å RMSD from the folded state. Some alignment between protein-specific models and GBn2 also occurs for certain RMSD ranges for BBA and NTL9. This alignment is likely the result of our pre-training and parameter freezing scheme. While we have demonstrated that this scheme is essential to prevent non-physical results, it is possible that freezing all of the interaction blocks results in too much of a loss of flexibility for some of the larger proteins in our training set.

To further investigate the effect of model flexibility, we trained several WW domain-specific models using the potential contrasting method while unfreezing 1 interaction block. Training was initialized with the same set of parameters obtained from pre-training the WW domain-specific model discussed in the previous paragraph. While all three of the resultant free energy profiles (Figure S4) are noisy to some extent, the model with the 1st interaction block (i.e., closest to the embedding layer and furthest from outputting the final updated atomic featurizations used as inputs to the energy-predicting NN) unfrozen most closely matches the explicit solvent free energy profile. Unlike the transferable model, all three

models trained with 1 interaction block unfrozen correctly capture the global free energy minimum observed in the explicit solvent results for WW domain. Interestingly, which of the 3 interaction blocks is unfrozen appears to affect accuracy. It is possible that by unfreezing the 1st of the 3 interaction blocks, some essential inflexibility is retained as the output of the unfrozen interaction block still must be fed through the remaining 2 frozen blocks. Overall, these results suggest that limitations to the flexibility of the SchNet architecture may result in the inaccuracy for WW domain and NTL9 observed in our transferable model (Figure 5).

Figures

All figures referenced in the main text (Figures S1 to S14) and all figures referenced exclusively in this Supporting Information document (Figures S15 to S23) are displayed below.

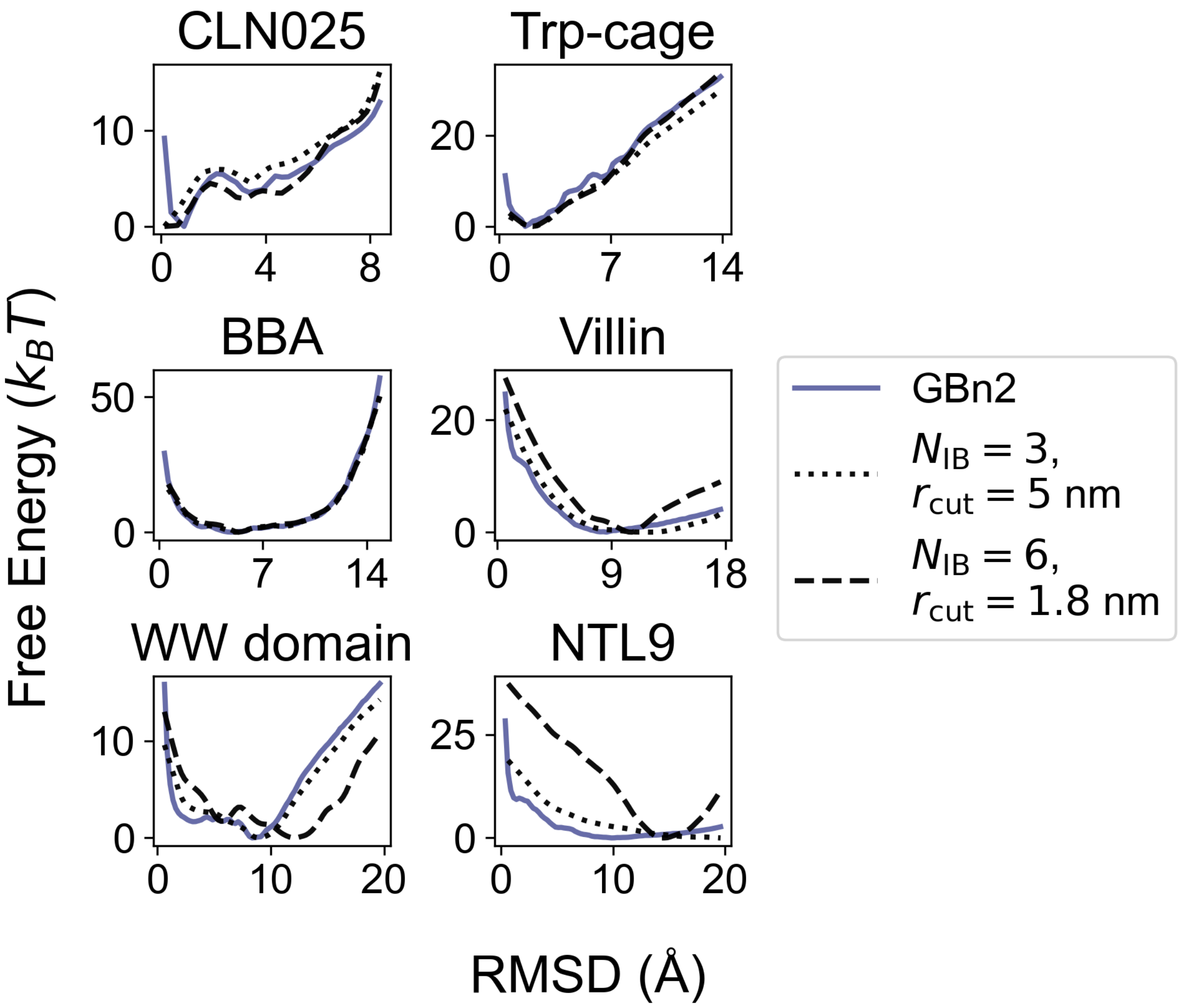

Figure S1: A SchNet model[S22] with larger r_{cut} but smaller N_{IB} outperforms a model with smaller r_{cut} but larger N_{IB}. Both models were trained to fit E_{GBn2}. Free energy profiles for the model with larger r_{cut} are shown in the dotted line, while free energy profiles for the model with a larger N_{IB} are shown in the dashed lines. For reference, the free energy profiles computed from umbrella simulations with the GBn2 implicit solvent model are also shown as solid lines. The x axes in the plots correspond to the RMSD from the folded structures. Results for the SchNet model were determined using a re-weighting scheme as detailed in the *Methods* section.

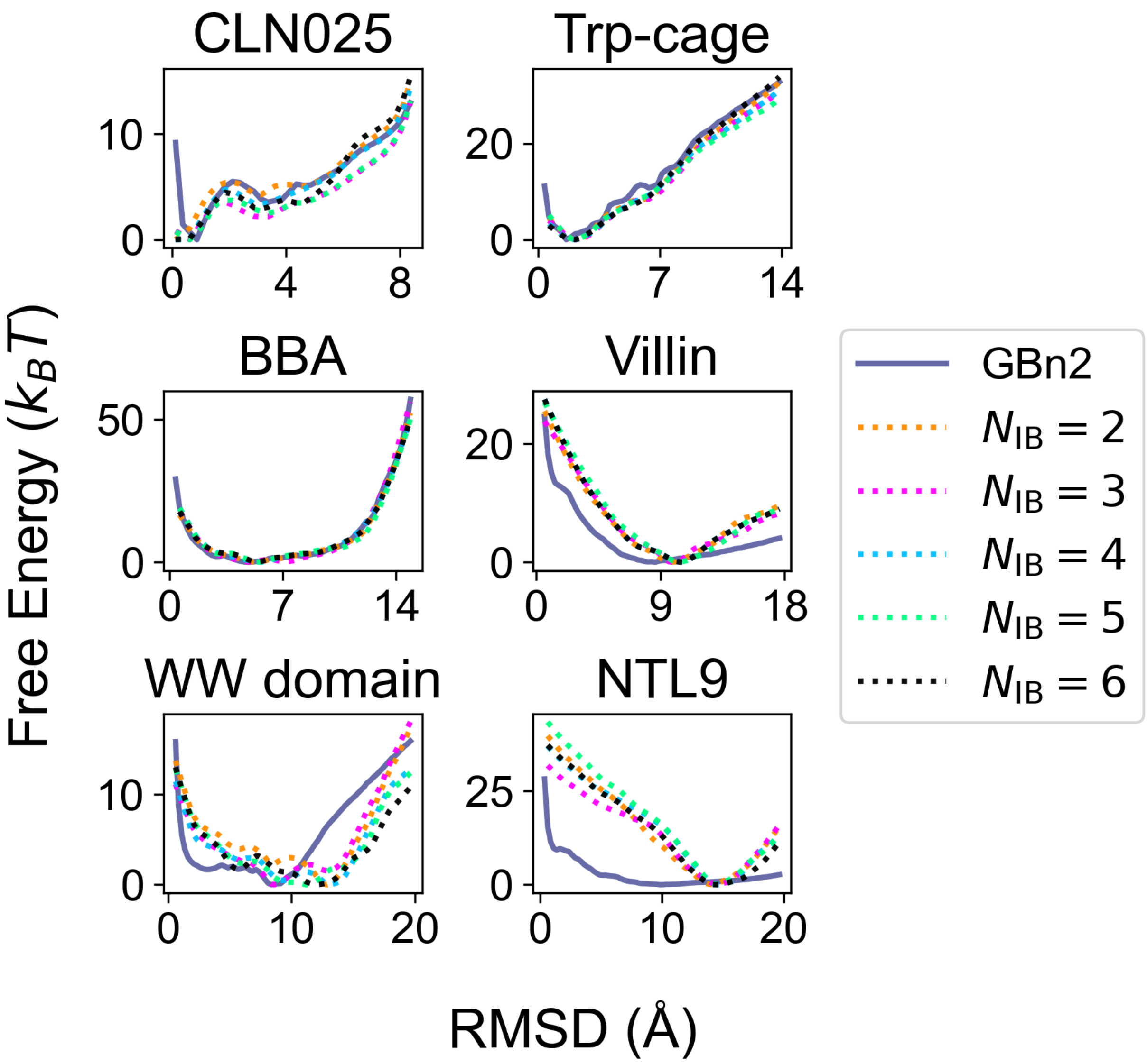

Figure S2: SchNet models trained to fit E_{GBn2} with different values of N_{IB} perform similarly. Free energy profiles are shown for all models. The cutoff distance is held constant at $r_{\mathrm{cut}} =$ 1.8 nm. The x axes in the plots correspond to the RMSD from the folded structures. For reference, the free energy profiles computed from umbrella simulations with the GBn2 implicit solvent model are also shown as solid lines. Results for the SchNet model were determined using a re-weighting scheme as detailed in the *Methods* section.

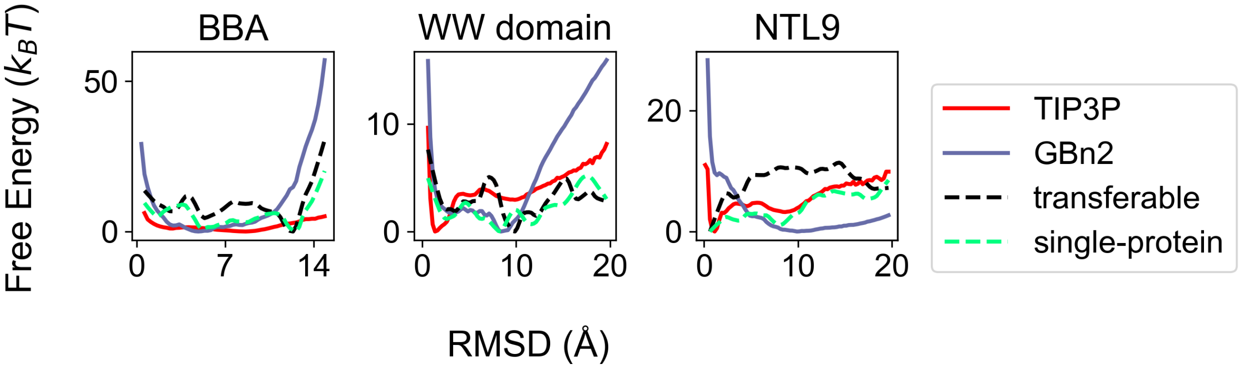

Figure S3: Re-weighted free energy profiles computed for protein-specific (denoted as "single-protein") SchNet models trained as implicit solvents with the potential contrasting method. Results for the SchNet models were determined using a re-weighting scheme as detailed in the *Methods* section. For reference, free energy profiles computed from configurations from TIP3P explicit solvent simulations conducted by Lindorff-Larsen et al. [S2], free energy profiles computed from umbrella simulations with the GBn2 implicit solvent, and free energy profiles for the transferable SchNet model (as shown in Figure 5, denoted here as "transferable") are displayed. The NTL9-specific model more closely matches the TIP3P results than the transferable model. Interestingly, from 0 to ∼10 Å RMSD, the WW domain single-protein free energy profile closely aligns with the GBn2 results. See section *Possible causes of lower accuracy for BBA, WW domain, & NTL9* for more details.

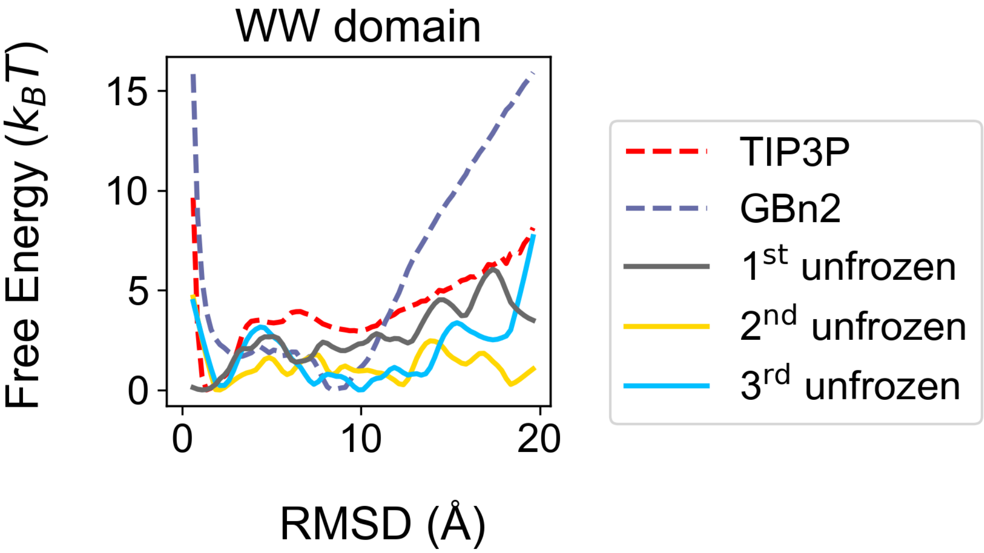

Figure S4: Re-weighted free energy profiles computed for WW domain-specific SchNet models trained with the potential contrasting method while unfreezing 1 interaction block. Results for the SchNet models were determined using a re-weighting scheme as detailed in the *Methods* section. For reference, the free energy profile computed from configurations from TIP3P explicit solvent simulations conducted by Lindorff-Larsen et al.[S2] and the free energy profile computed from umbrella simulations with the GBn2 implicit solvent are displayed. While all models correctly capture the results around the TIP3P global minimum at ∼1 Å RMSD from the folded state, the model trained with the 1st interaction block unfrozen most closely matches the TIP3P results. See section *Possible causes of lower accuracy for BBA, WW domain, & NTL9* for more details.

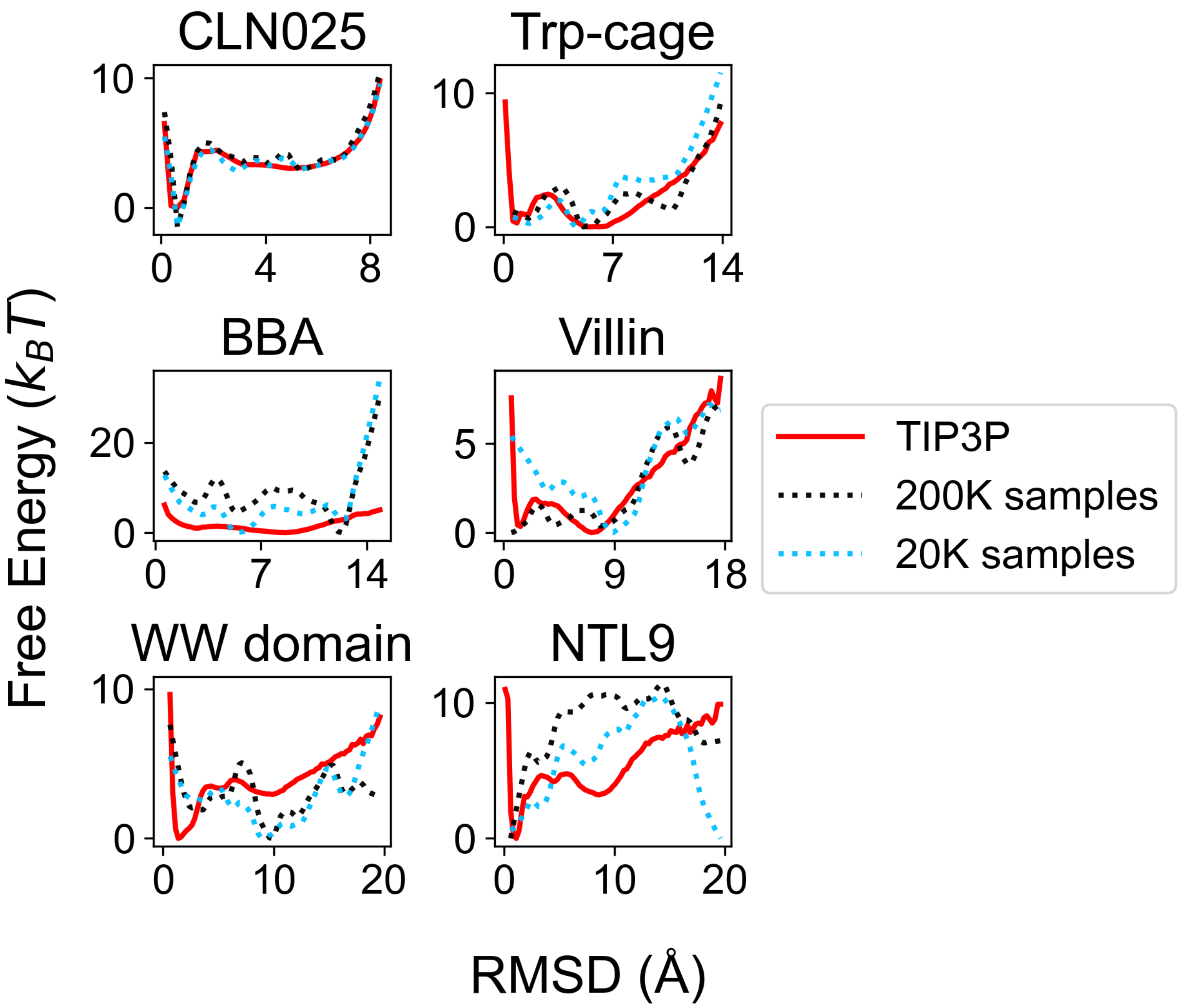

Figure S5: Free energy profiles for SchNet models ($r_{\text{cut}} = 5$ nm, $N_{\text{IB}} = 3$) trained with the potential contrasting method with different amounts of input data. The free energy profiles computed from simulations by Lindorff-Larsen et al.[S2] in TIP3P explicit solvent are shown in red for comparison. Results for the SchNet models were determined using a re-weighting scheme as detailed in the *Methods* section. The free energy profiles for the model denoted as "200K samples" are the same as those displayed in Figure 5. From the original training data of 200,000 configurations per protein, every 10th configuration was selected for use as training data for the data-limited model. The free energy profiles for the data-limited model are denoted as "20K samples". Due to the sharpness of the minimum in the SchNet free energy profiles for CLN025, the free energy profile was uniformly shifted downwards by 1.5 $k_B T$ for better visualization. Despite training with less data, all noise configurations were used to compute the free energy profiles for the data-limited model. The free energy profiles generated from the data-limited model are similar to those generated from the original model without data limitations, with the exception of NTL9.

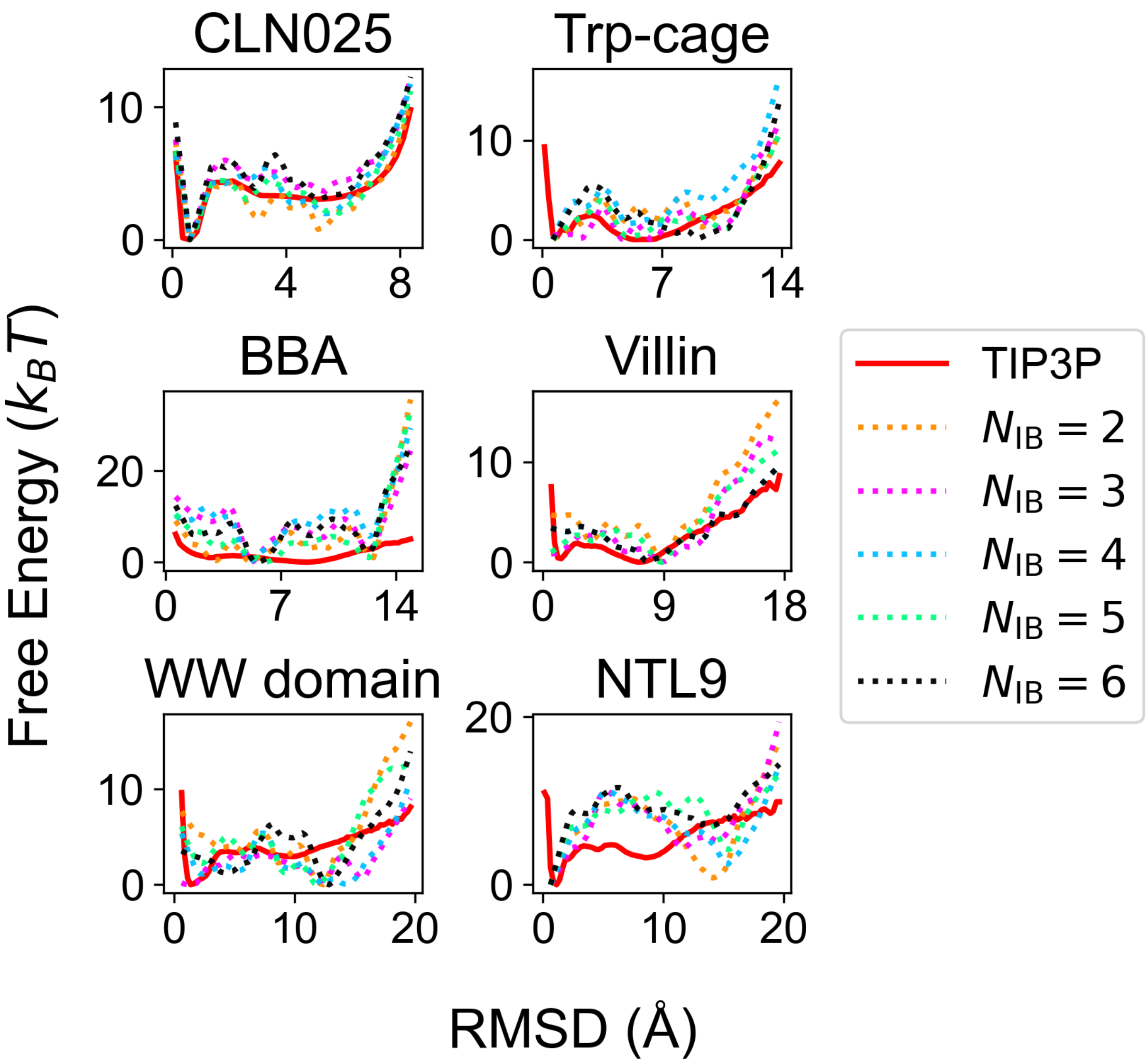

Figure S6: Free energy profiles for SchNet models trained with potential contrasting with different values of N_{IB}. The cutoff distance is held constant at $r_{\mathrm{cut}} = 1.8$ nm. The x axes in the plots correspond to the RMSD from the folded structures. For reference, the free energy profiles computed from simulations conducted by Lindorff-Larsen et al.[S2] in TIP3P explicit solvent are shown as red solid lines. Results for the SchNet model were determined using a re-weighting scheme as detailed in the *Methods* section.

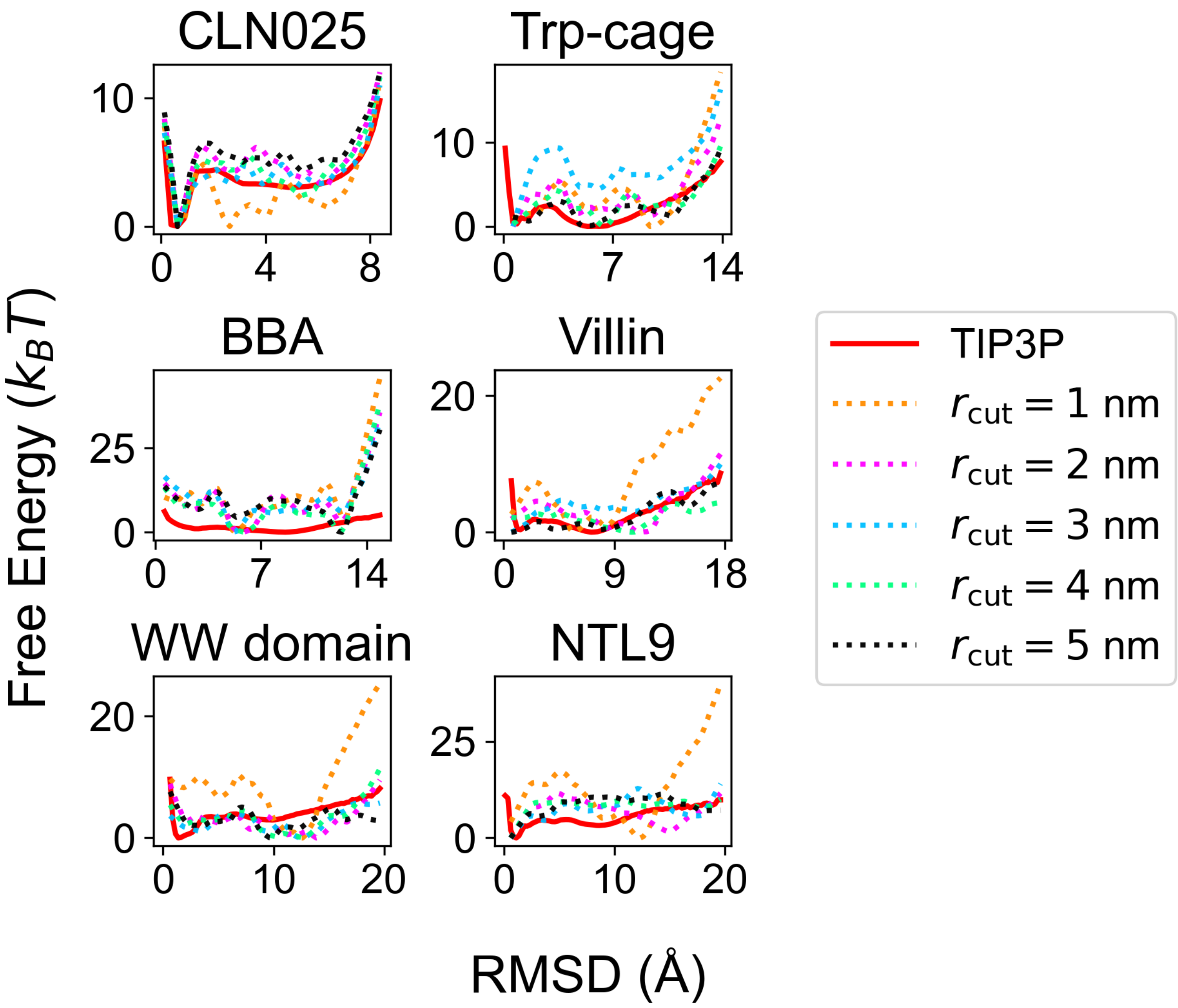

Figure S7: Free energy profiles for SchNet models trained with potential contrasting with different values of r_{cut}. The number of interaction blocks is held constant at $N_{\mathrm{IB}} = 3$. The x axes in the plots correspond to the RMSD from the folded structures. For reference, the free energy profiles computed from simulations conducted by Lindorff-Larsen et al.[S2] in TIP3P explicit solvent are shown as red solid lines. Results for the SchNet models were determined using a re-weighting scheme as detailed in the *Methods* section.

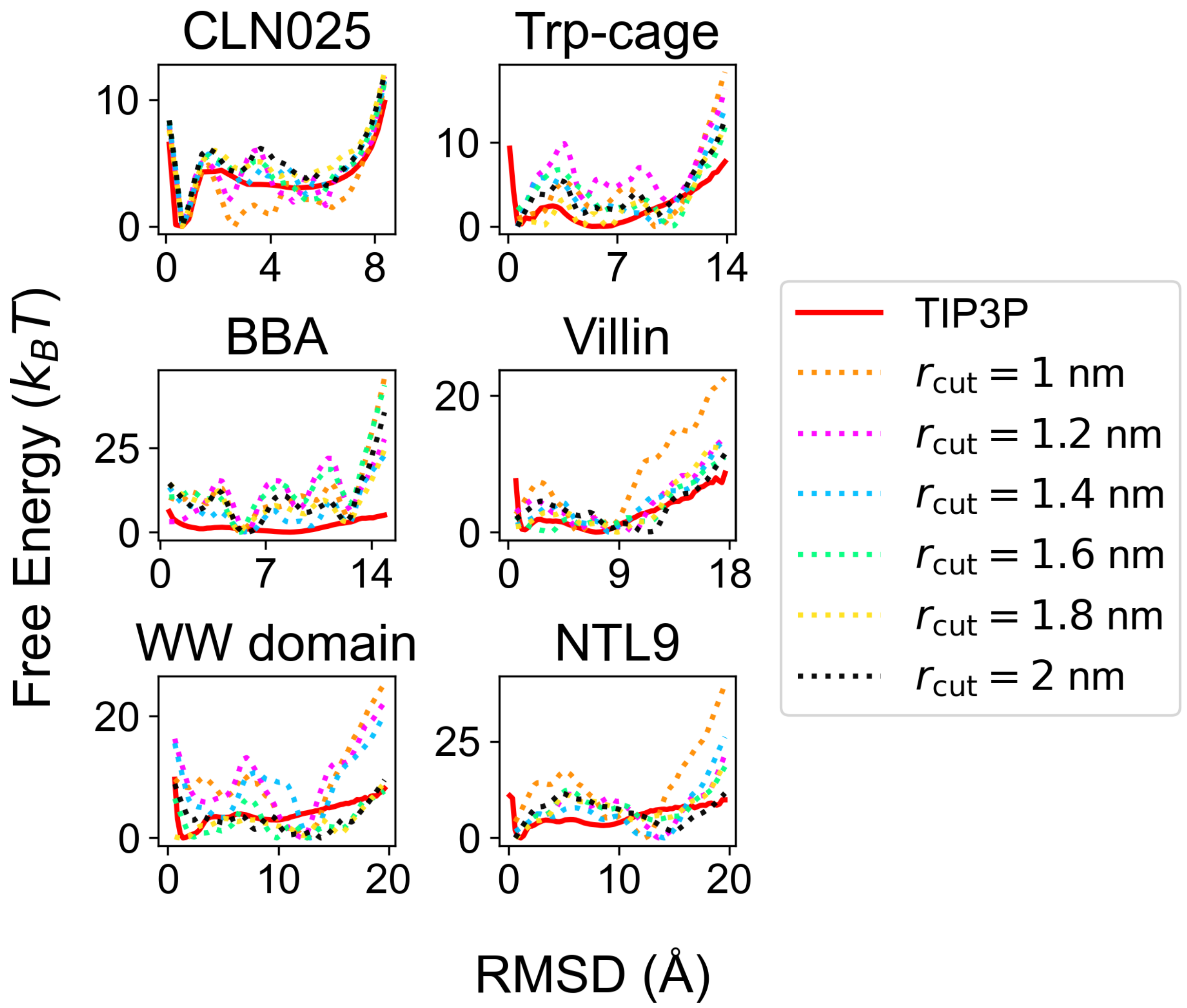

Figure S8: Free energy profiles for SchNet models trained with potential contrasting with values of r_{cut} from 1 to 2 nm. The number of interaction blocks is held constant at $N_{\text{IB}} = 3$. The x axes in the plots correspond to the RMSD from the folded structures. For reference, the free energy profiles computed from simulations conducted by Lindorff-Larsen et al.[S2] in TIP3P explicit solvent are shown as red solid lines. Results for the SchNet models were determined using a re-weighting scheme as detailed in the *Methods* section.

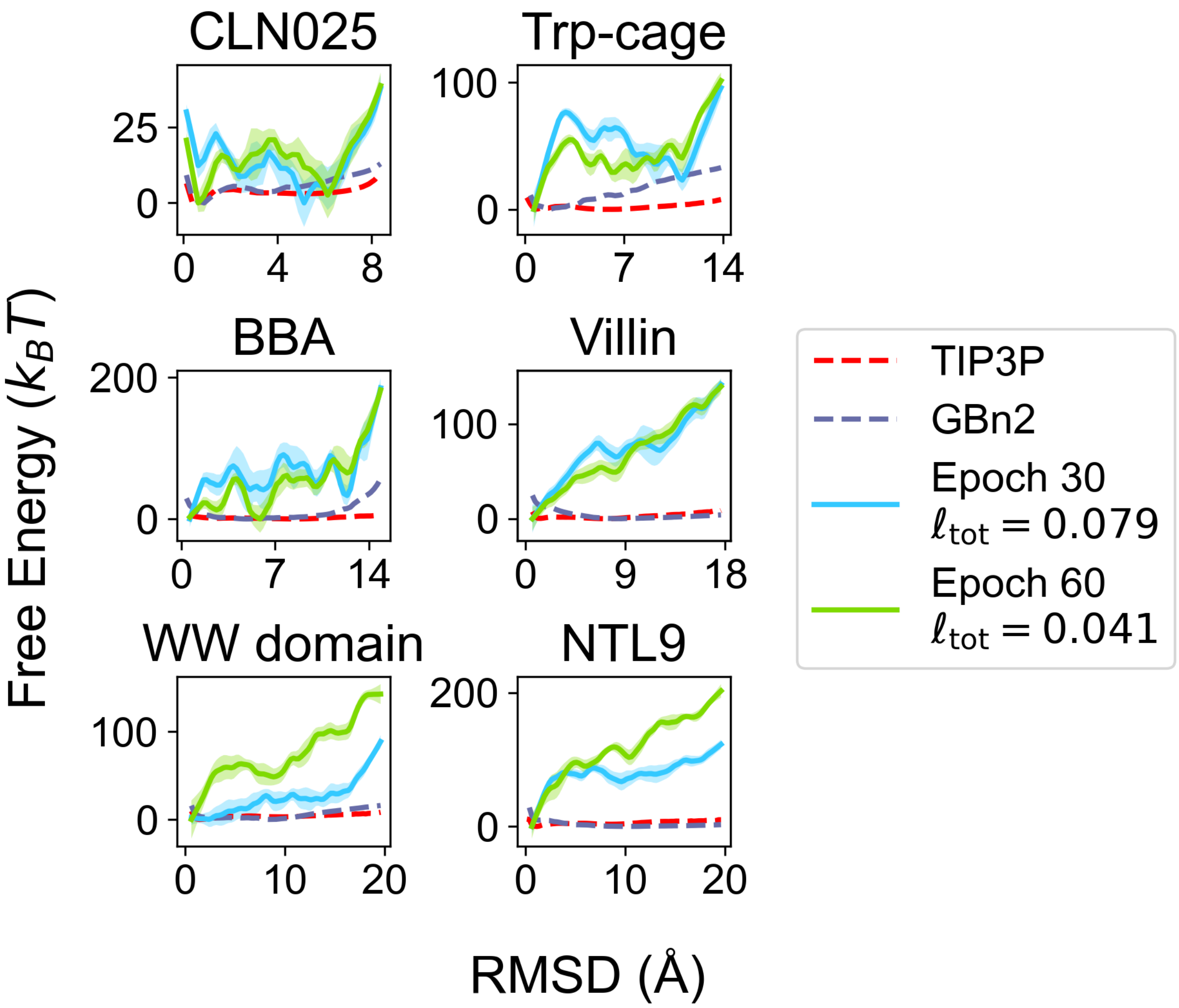

Figure S9: A SchNet model ($r_{cut} = 1.8$ nm, $N_{IB} = 3$) trained with the potential contrasting method without prior pre-training fails to match the free energy profiles from explicit solvent simulations. Free energy profiles for the model trained for 30 and 60 epochs are shown in light blue and light green, respectively. The x axes in the plots correspond to the RMSD from the folded structures. Performance is still poor despite the model trained for 60 epochs reaching a similar value of the potential contrasting loss function ($\ell_{tot} = 0.041$) to the model used for ML-MD simulations in the main text ($r_{cut} = 1.8$ nm, $N_{IB} = 3$; $\ell_{tot} = 0.038$). For reference, the free energy profiles computed from simulations conducted by Lindorff-Larsen et al.[S2] in TIP3P explicit solvent are shown as red dashed lines, and the free energy profiles computed from umbrella simulations with the GBn2 implicit solvent model are shown as blue dashed lines. Results for the SchNet model were determined using a re-weighting scheme as detailed in the *Methods* section.

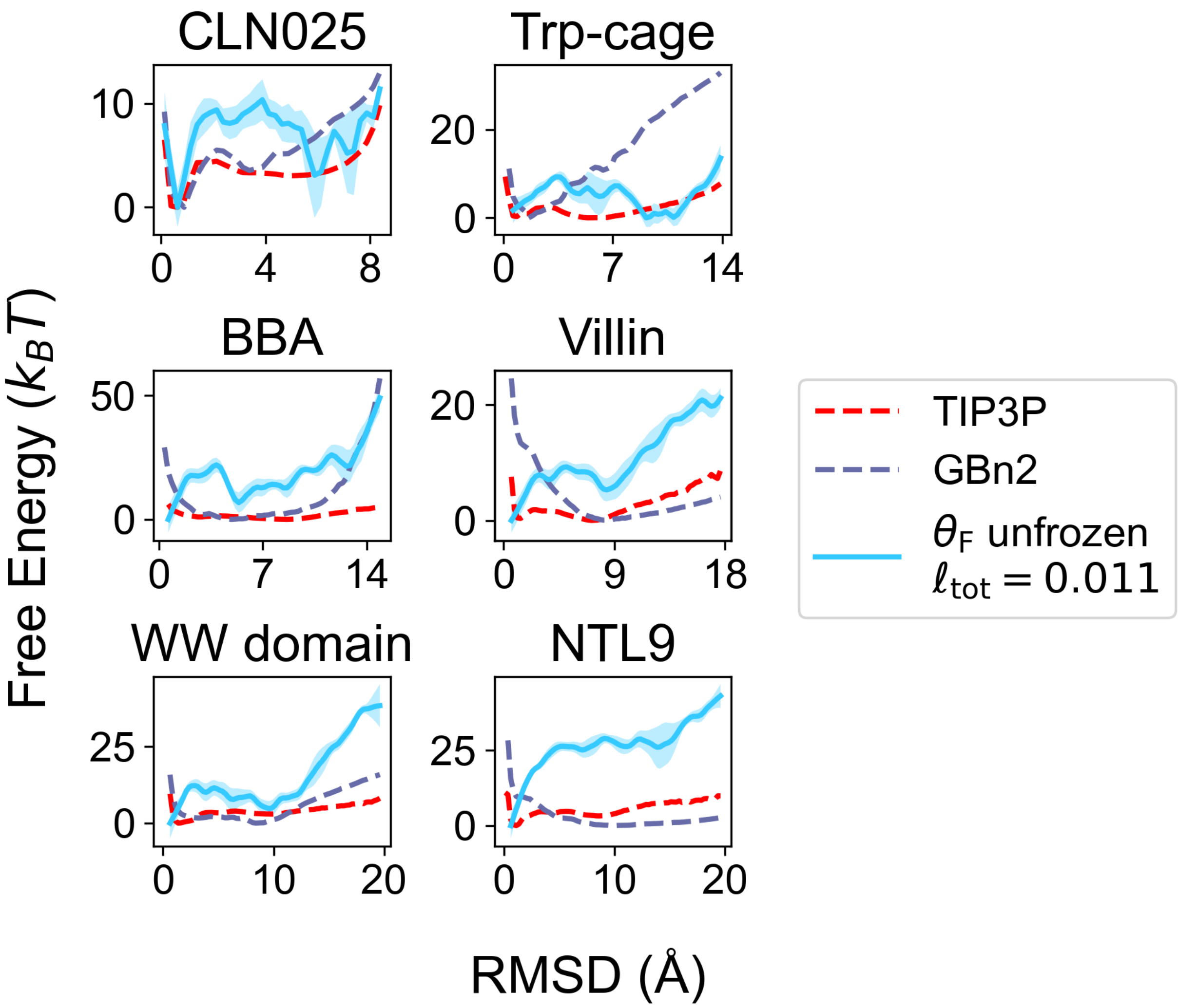

Figure S10: A SchNet model ($r_{\rm cut} = 1.8$ nm, $N_{\rm IB} = 3$) trained with the potential contrasting method and with prior pre-training, but without holding the interaction block parameters ($\boldsymbol{\theta}_{\mathbf{F}}$) constant, fails to match the free energy profiles from explicit solvent simulations. The free energy profiles for this model are shown in light blue. The x axes in the plots correspond to the RMSD from the folded structures. Notably, this model reaches a lower value of the potential contrasting loss function ($\ell_{\rm tot} = 0.11$) than the model used for ML-MD simulations in main text ($r_{\rm cut} = 1.8$ nm, $N_{\rm IB} = 3$; $\ell_{\rm tot} = 0.038$). For reference, the free energy profiles computed from simulations conducted by Lindorff-Larsen et al.[S2] in TIP3P explicit solvent are shown as red dashed lines, and the free energy profiles computed from umbrella simulations with the GBn2 implicit solvent model are shown as blue dashed lines. Results for the SchNet model were determined using a re-weighting scheme as detailed in the *Methods* section.

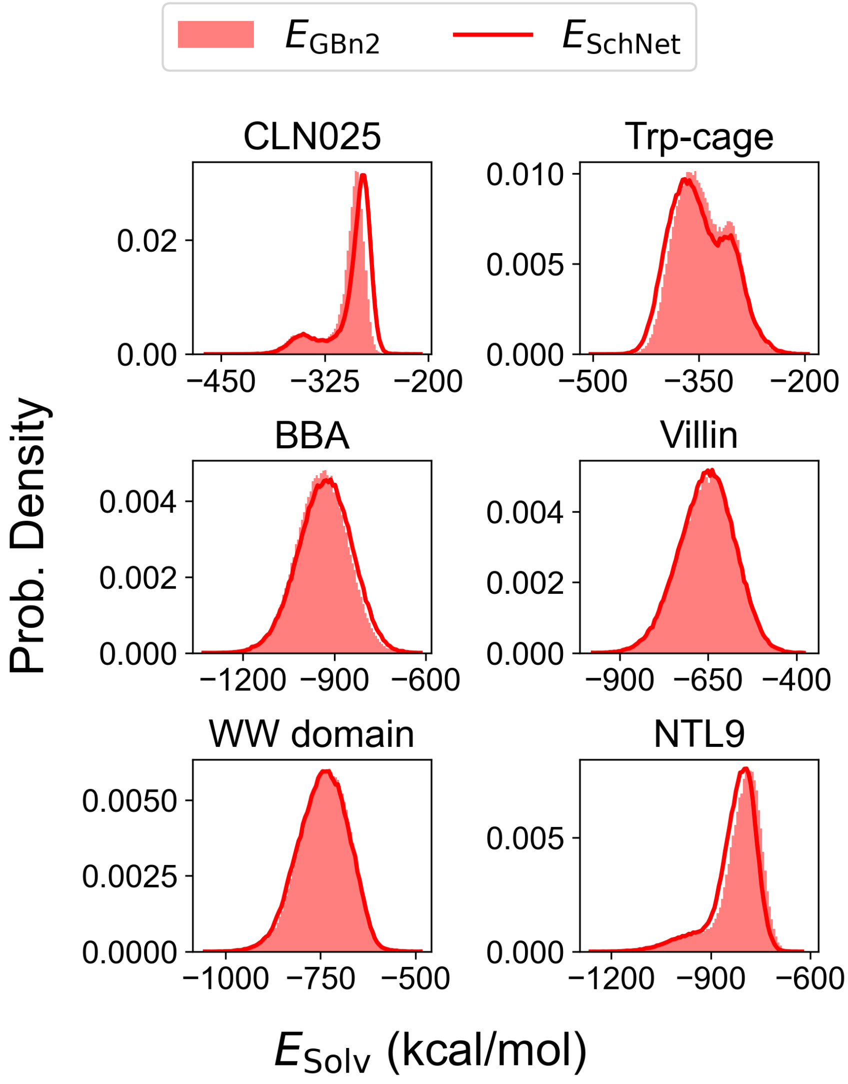

Figure S11: The optimal SchNet model predicts solvation free energy (E_{SchNet}) distributions that are similar in scale to the GBn2 solvation free energy distributions (E_{GBn2}). Solvation free energies are shown for a SchNet model with $r_{\mathrm{cut}} = 1.8$ nm and $N_{\mathrm{IB}} = 3$. The E_{GBn2} distributions are displayed as filled in, while the E_{SchNet} distributions are shown as solid lines.

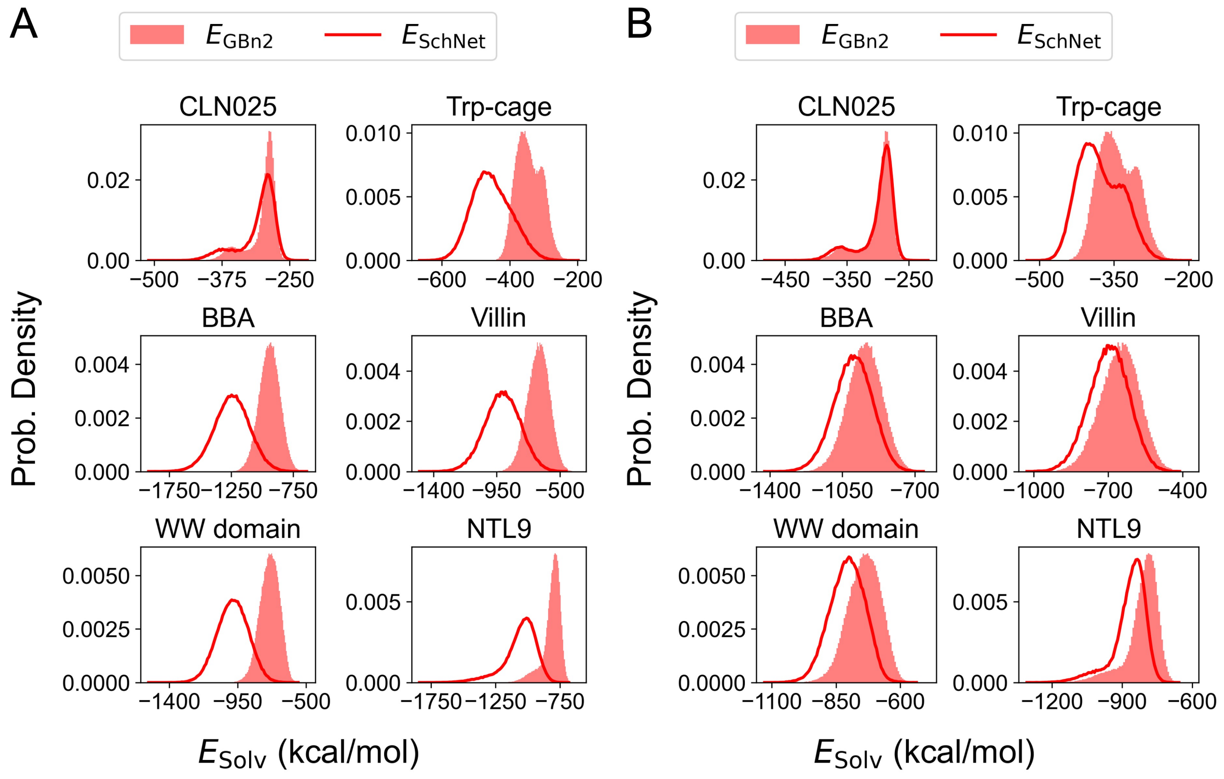

Figure S12: A SchNet implicit solvent trained without pre-training or without holding the interaction block constant yields unrealistic solvation free energies (E_{SchNet}). Solvation free energies are shown for a SchNet model with $r_{\mathrm{cut}} = 1.8$ nm and $N_{\mathrm{IB}} = 3$. The E_{GBn2} distributions are displayed as filled in, while the E_{SchNet} distributions are shown as solid lines. (A) E_{SchNet} distributions for a model that was not pre-trained. For most proteins, SchNet appears to arbitrarily predict low energies. (B) E_{SchNet} distributions for a model that was pre-trained, but did not have the interaction blocks held constant during training with potential contrasting. Compared to the distributions shown in Figure S11, these distributions are shifted more negatively.

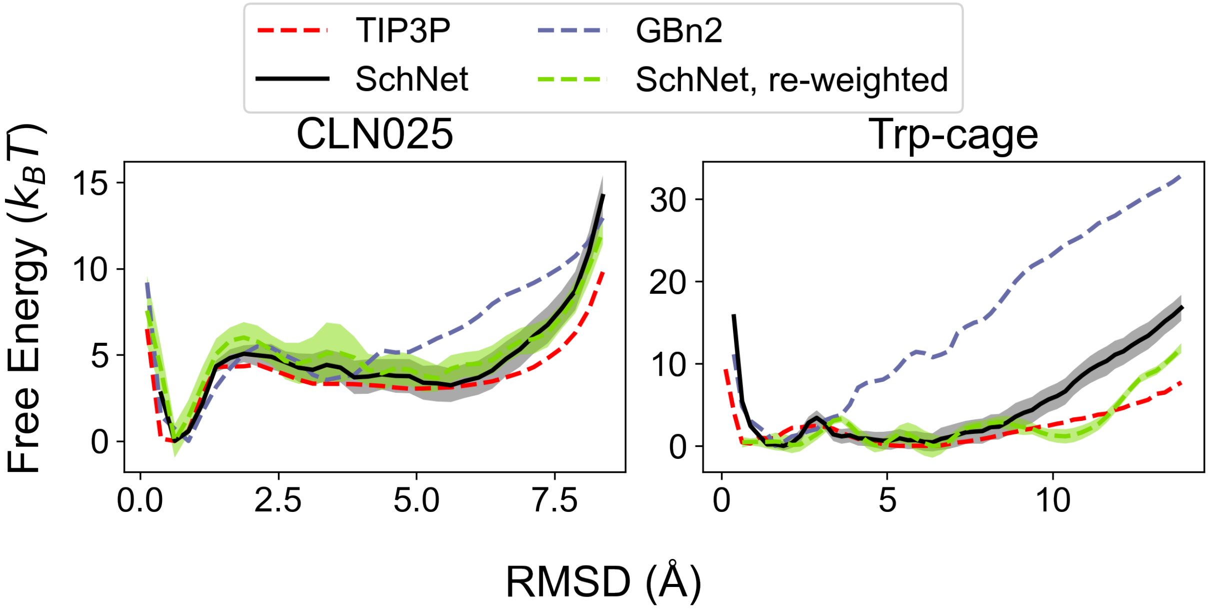

Figure S13: Re-weighted free energy profiles closely match those computed from ML-MD simulations. Explicit solvent (denoted as TIP3P), GBn2, and the SchNet implicit solvent free energy profiles (both re-weighted and from ML-MD simulations) for chignolin CLN025 and Trp-cage are displayed. The x axes in the plots correspond to the RMSD from the folded structures. TIP3P free energy profiles were computed from the configurations generated by Lindorff-Larsen et al.[S2] while GBn2 and SchNet free energy profiles were computed by solving the MBAR equation for configurations from umbrella sampling simulations. The re-weighted SchNet free energy profiles ($r_{\mathrm{cut}} = 1.8$ nm, $N_{\mathrm{IB}} = 3$) were computed following the re-weighting procedure detailed in the *Methods* section.

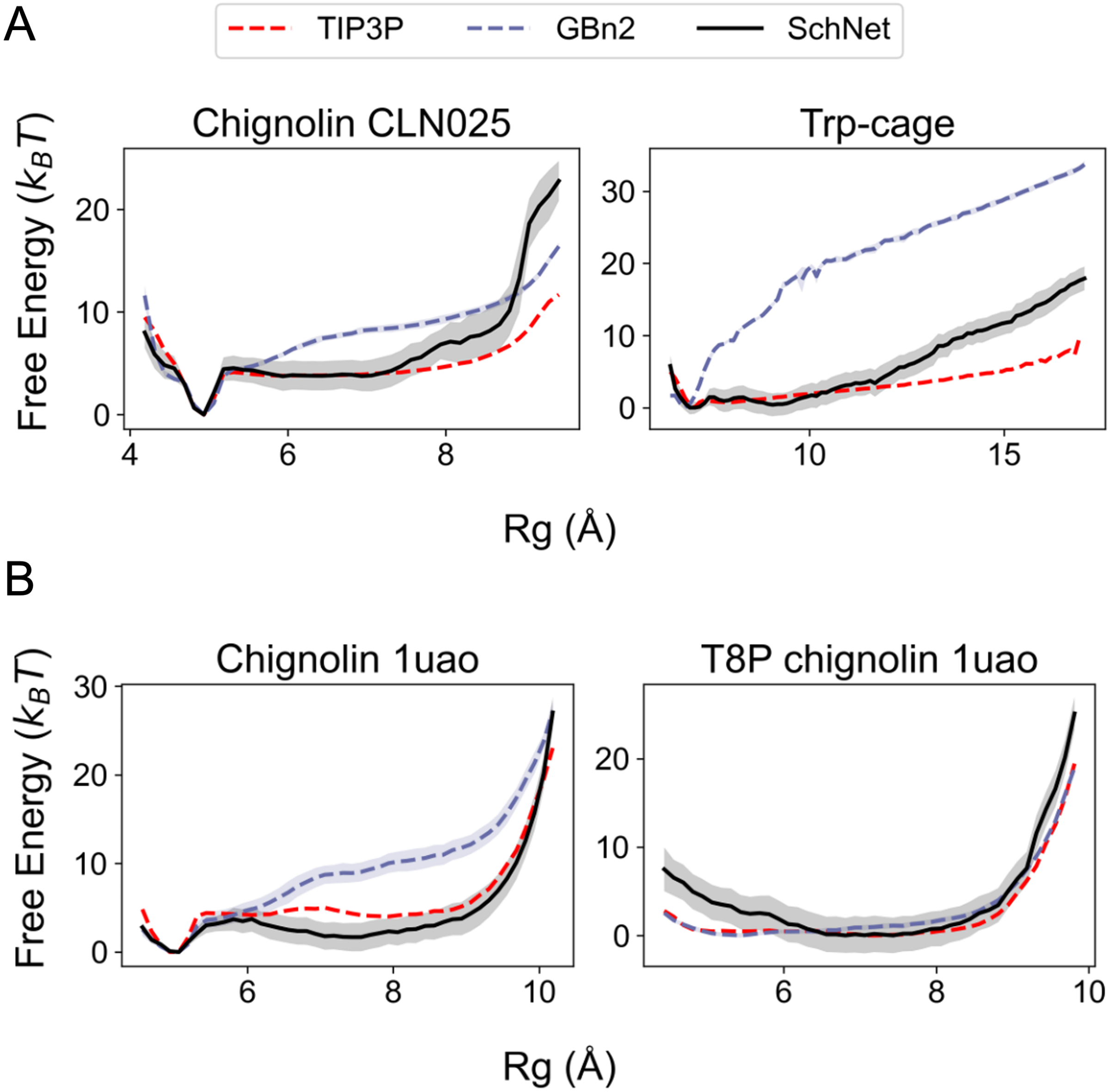

Figure S14: Comparison between the free energy profiles computed from explicit solvent simulations (TIP3P), implicit solvent simulations (GBn2), and the optimized SchNet model. Results for the SchNet model were determined using ML-MD umbrella simulations. The x axes in the plots correspond to the radius of gyration (Rg) of the backbone heavy atoms. (A) Free energy profiles for chignolin CLN025 and Trp-cage. (B) Free energy profiles for chignolin 1uao and a T8P mutant. TIP3P and GBn2 free energy profiles were computed from umbrella simulations.

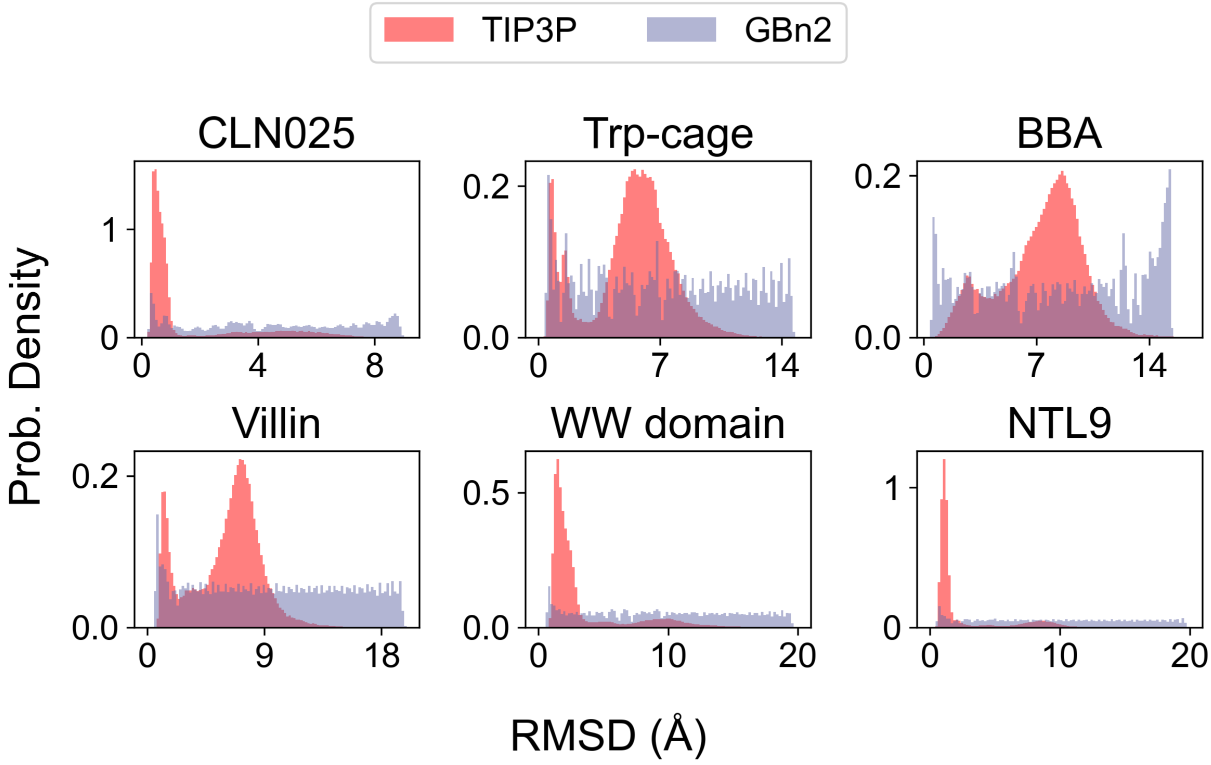

Figure S15: Explicit solvent (denoted as TIP3P) and GBn2 configuration RMSD histograms. RMSD is computed with respect to peptide backbone heavy atom positions from $\boldsymbol{x}_{\circ}$. All TIP3P configurations for these 6 proteins were generated by Lindorff-Larsen et al.[S2] Noise configurations from the GBn2 umbrella sampling ensembles fully cover the RMSD ranges sampled by the TIP3P data configurations, while also including configurations outside of these ranges. This follows best-practice for generating noise samples for training with potential contrasting.[S10]

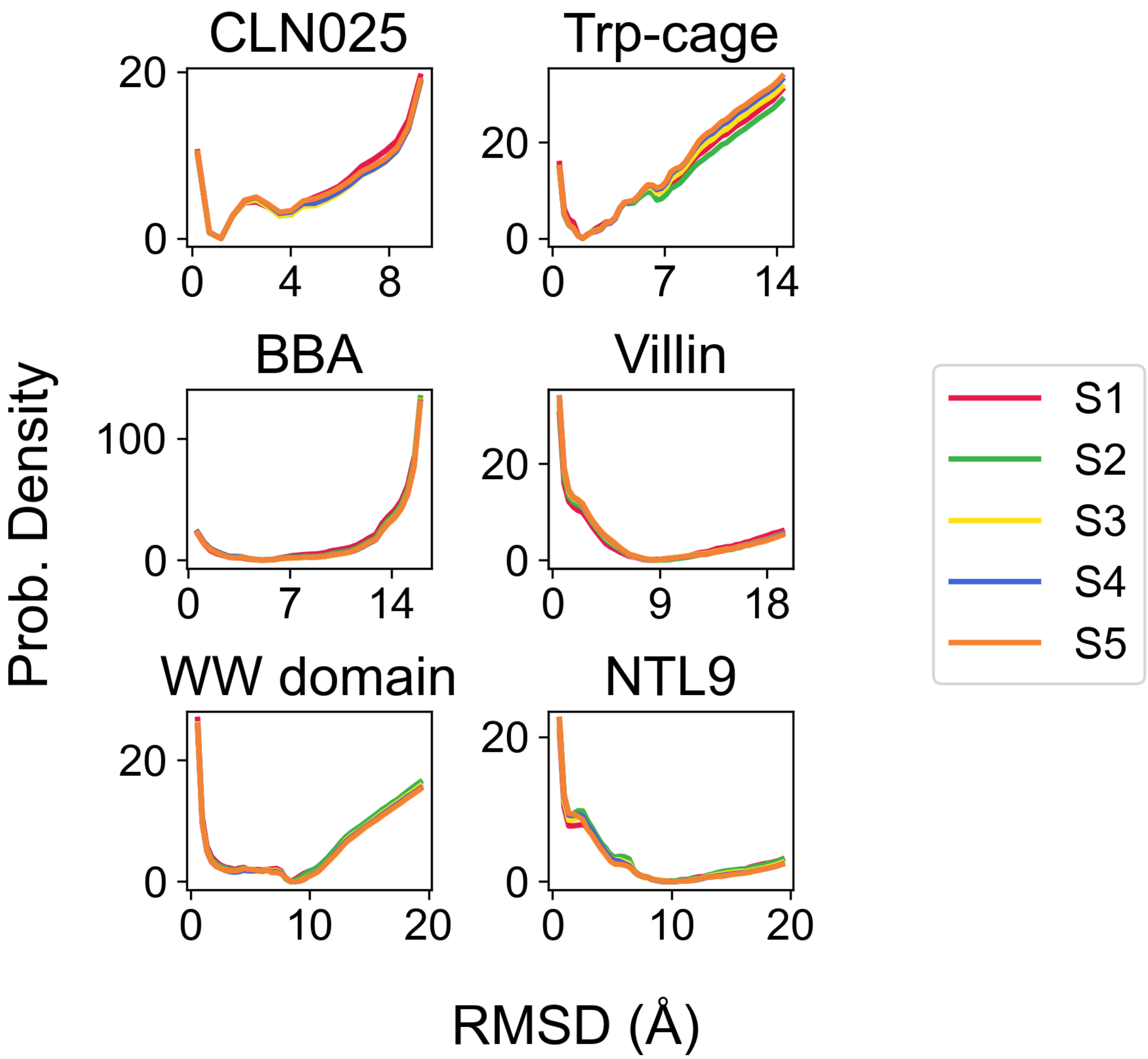

Figure S16: Converged free energy profiles for all training set proteins. Umbrella sampling windows were split 5 ways to show that umbrella sampling GBn2 free energy profiles were converged. These splits were created according the following example for CLN025: S1 0 - 212 ns, S2 0 - 424 ns, S3 0 - 636 ns, S4 0 - 848 ns, and S5 0 - 1,060 ns. For all proteins, the final split includes all configurations for each window.

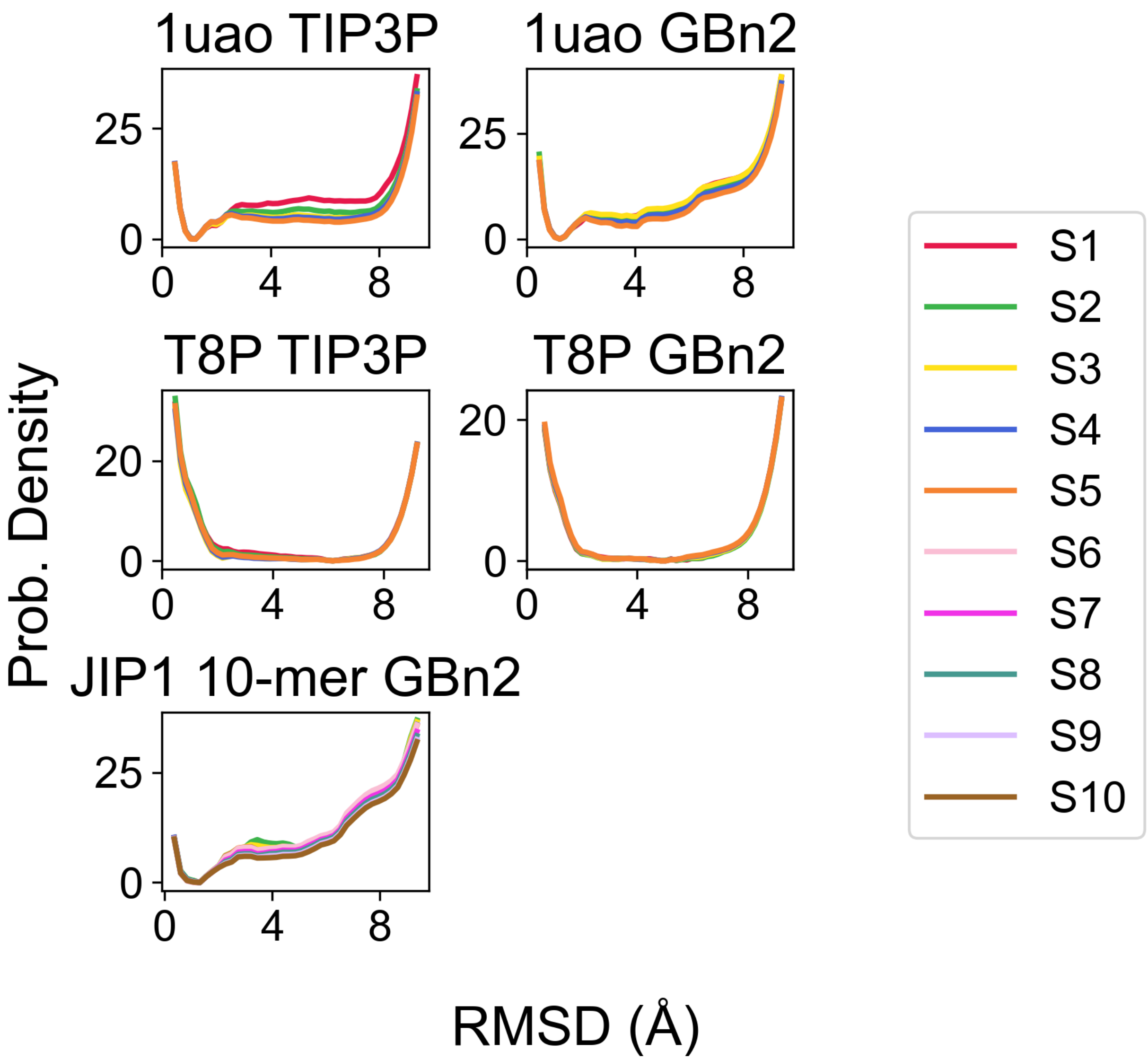

Figure S17: Converged free energy profiles for all testing set proteins. Umbrella sampling windows were split 5 ways for 1uao and T8P and 10 ways for the JIP1 10-mer to show that umbrella sampling TIP3P and GBn2 free energy profiles were converged. For 1uao and T8P, splits were created according the following scheme: S1 0 - 12.5 ns, S2 0 - 25 ns, S3 0 - 37.5 ns, S4 0 - 50 ns, and S5 0 - 62.5 ns. For the JIP1 10-mer, splits were created according to the following scheme: S1 0 - 100 ns, S2 0 - 200 ns, S3 0 - 300 ns, S4 0 - 400 ns, S5 0 - 500 ns, S6 0 - 600 ns, S7 0 - 700 ns, S8 0 - 800 ns, S9 0 - 900 ns, and S10 0 - 1,000 ns. For all proteins, the final split includes all configurations for each window.

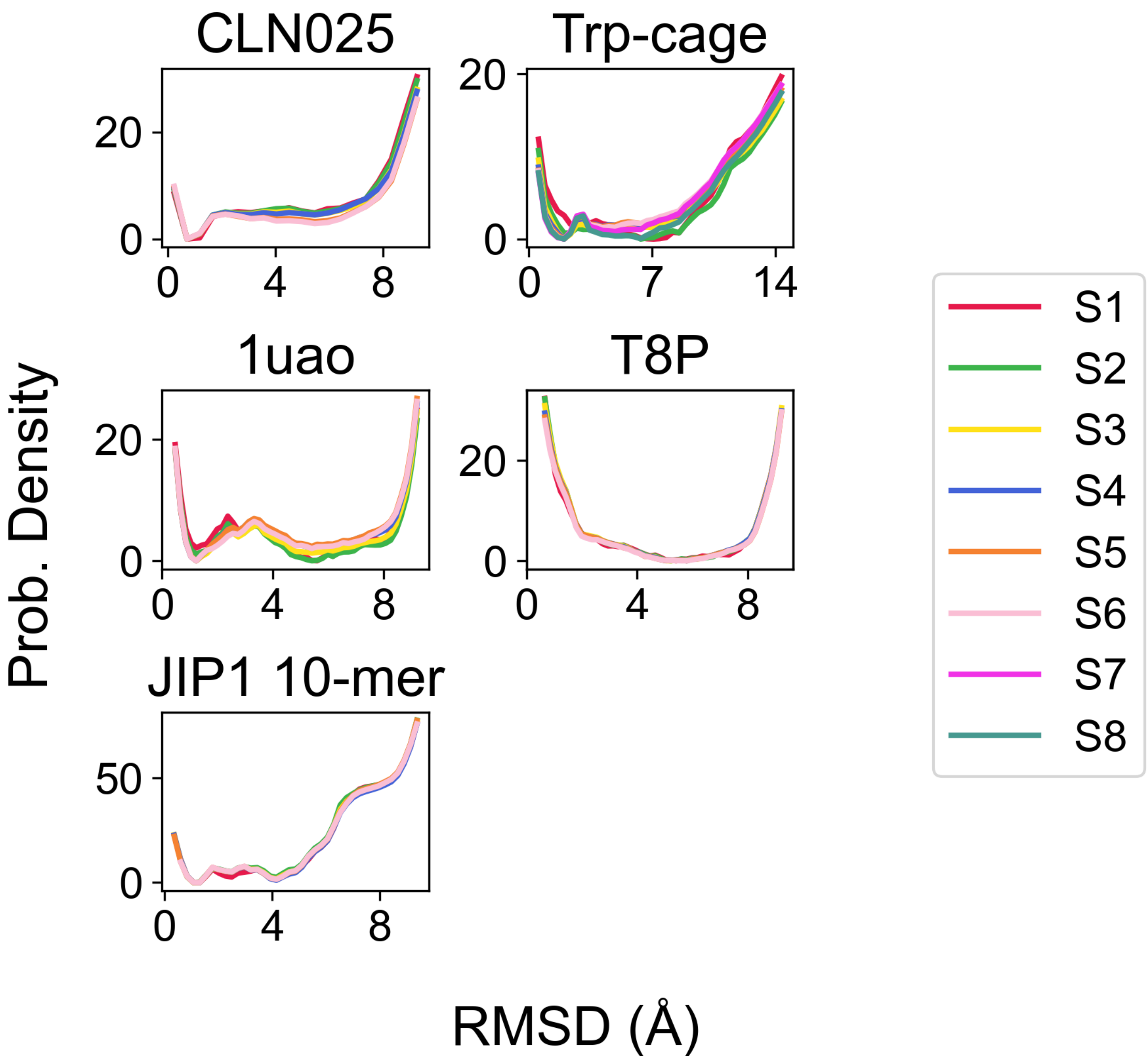

Figure S18: Converged free energy profiles for all proteins used to evaluate the SchNet implicit solvent model. Umbrella sampling windows were split 5 ways to show that the free energy profiles were converged. For CLN025, 1uao, and T8P, splits were created according the following scheme: S1 0 - 2 ns, S2 0 - 4 ns, S3 0 - 6 ns, S4 0 - 8 ns, S5 0 - 10 ns, and S6 0 - 13 ns. For the JIP1 10-mer, splits were created according to the following scheme: S1 0 - 5 ns, S2 0 - 10 ns, S3 0 - 15 ns, S4 0 - 20 ns, S5 0 - 25 ns, and S6 0 - 30 ns. For Trp-cage, splits were created according to the following scheme: S1 0 - 2.5 ns, S2 0 - 5 ns, S3 0 - 7.5 ns, S4 0 - 10 ns, S5 0 - 12.5 ns, S6 0 - 15 ns, S7 0 - 17.5 ns, and S8 0 - 20 ns. For all proteins, the final split includes all configurations for each window.

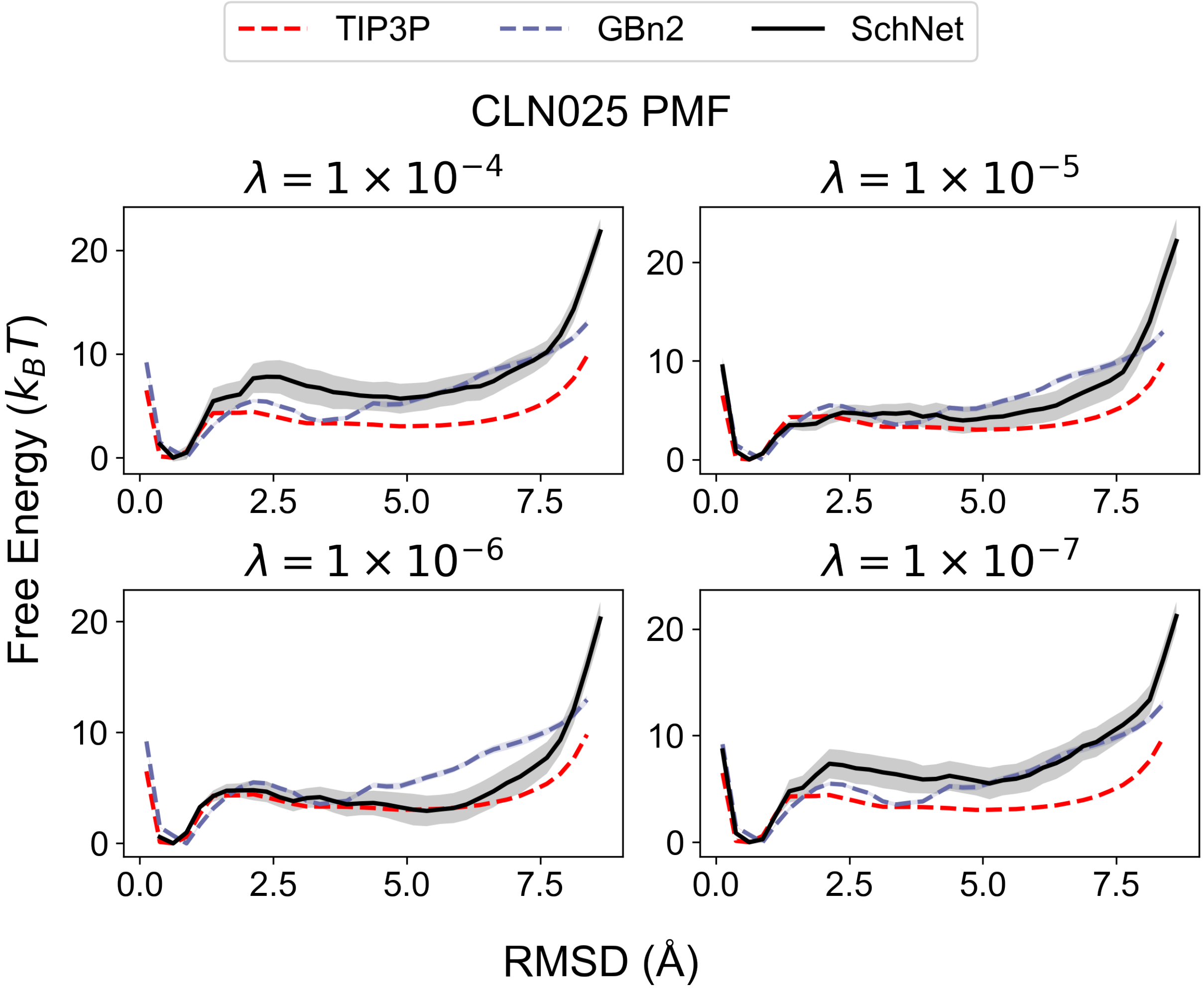

Figure S19: Impact of the L2 regularization constants, λ, on the performance of SchNet models ($N_{\mathrm{IB}} = 3$, $r_{\mathrm{cut}} = 1.8$ nm) trained with potential contrasting. We used the protein CLN025 as an example. Parameters in the interaction blocks were frozen during optimization with potential contrasting. The explicit solvent (TIP3P) free energy profile was computed from the configurations generated by Lindorff-Larsen et al.[S2], while configurations from umbrella sampling simulations were used to compute the GBn2 and SchNet free energy profiles. The SchNet model trained with $\lambda = 1 \times 10^{-6}$ produces a free energy profile that closely matches the explicit solvent result. See text *Section: Hyperparameters* for further discussion.

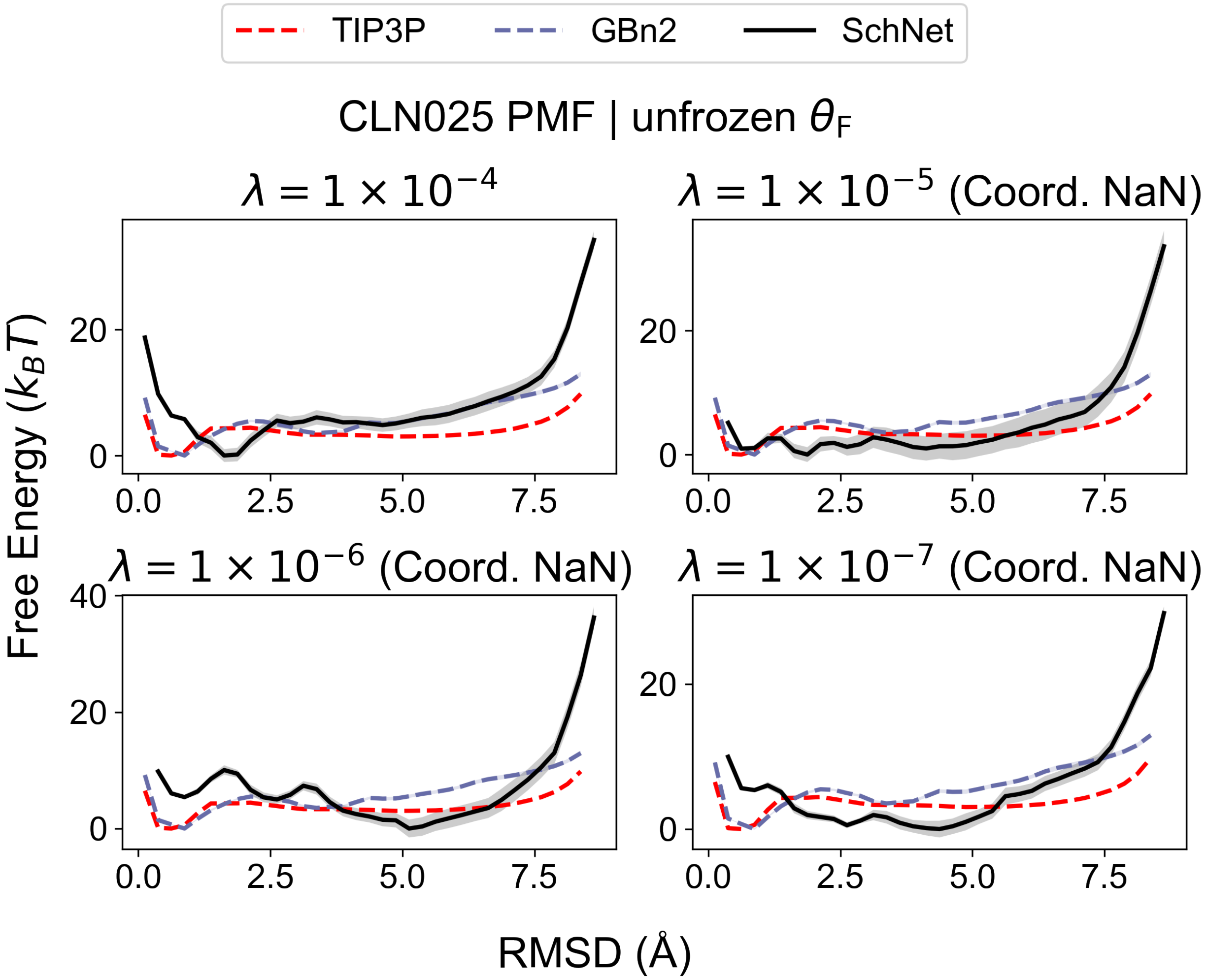

Figure S20: SchNet models ($N_{\text{IB}} = 3$, $r_{\text{cut}} = 1.8$ nm) trained using potential contrasting without freezing interaction block parameters performed poorly. We used the protein CLN025 as an example. The explicit solvent (TIP3P) free energy profiles was computed from the configurations generated by Lindorff-Larsen et al.[S2], while configurations from umbrella sampling simulations were used to compute the GBn2 and SchNet free energy profiles. No λ resulted in a SchNet free energy profile that closely matched the explicit solvent result. SchNet models with $\lambda < 1 \times 10^{-4}$ result in simulation instability.

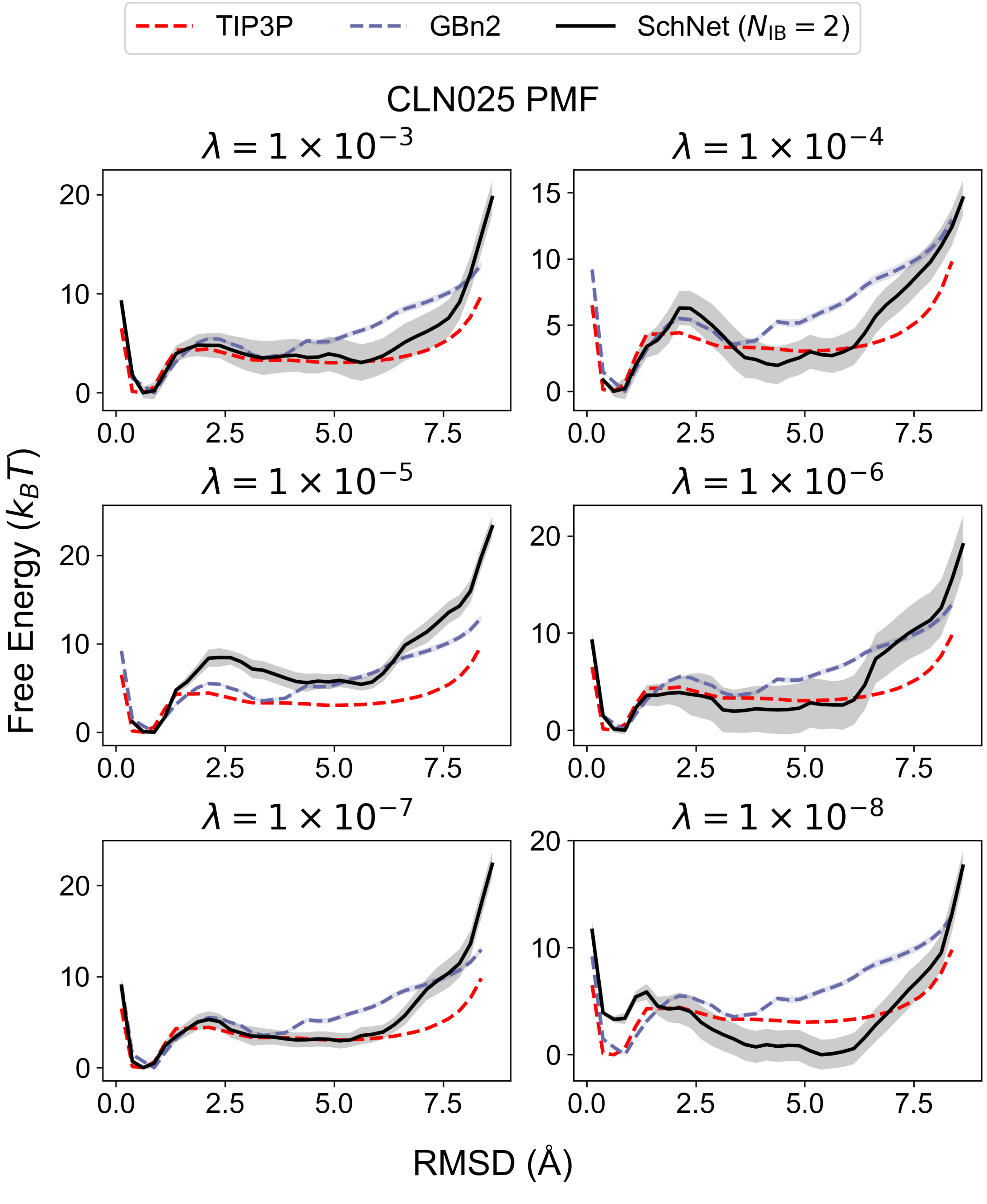

Figure S21: Impact of the L2 regularization constants, λ, on the performance of SchNet models ($N_{\rm IB} = 2$, $r_{\rm cut} = 1.8$ nm) trained with potential contrasting. We used the protein CLN025 as an example. The explicit solvent (TIP3P) free energy profiles was computed from the configurations generated by Lindorff-Larsen et al.[S2], while configurations from umbrella sampling simulations were used to compute the GBn2 and SchNet free energy profiles. The SchNet model with $\lambda = 1 \times 10^{-3}$ results in a free energy profile that most closely matches the explicit solvent result.

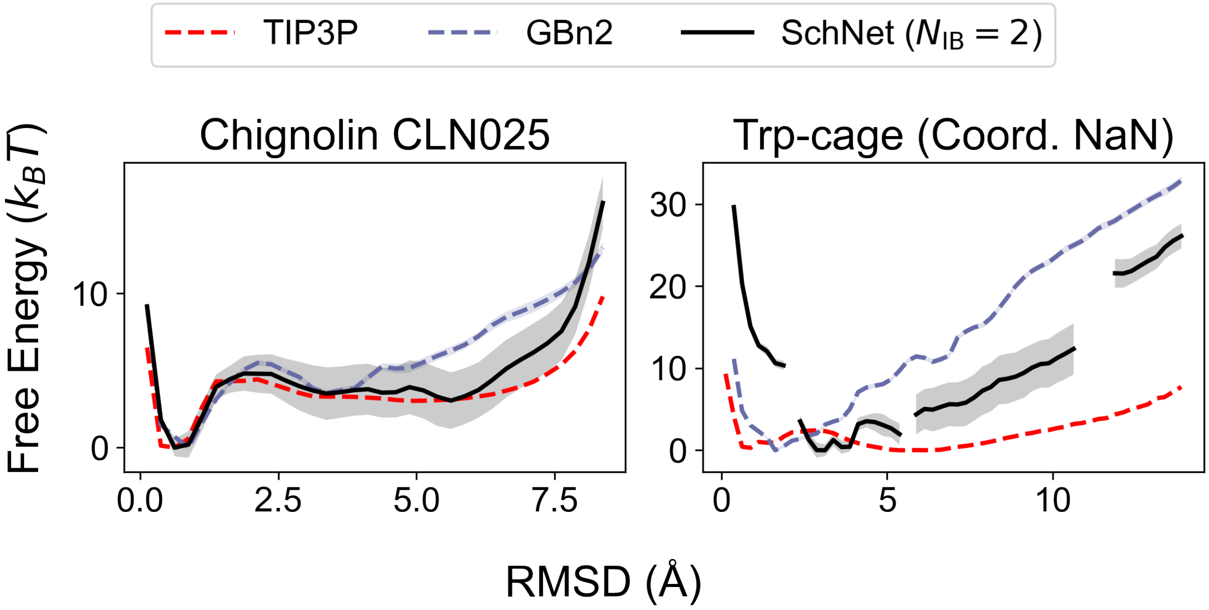

Figure S22: Free energy profiles for CLN025 and Trp-cage using a SchNet implicit solvent model with a smaller number of interaction blocks ($N_{\mathrm{IB}} = 2$, $r_{\mathrm{cut}} = 1.8$ nm, $\lambda = 1 \times 10^{-3}$). All explicit solvent (denoted as TIP3P) free energy profiles were computed from the configurations generated by Lindorff-Larsen et al.[S2], while configurations from umbrella sampling simulations were used to compute the GBn2 and SchNet free energy profiles. The SchNet free energy profile for CLN025 more closely matches the TIP3P free energy profile than the GBn2 free energy profile. Simulations for Trp-cage were too unstable to continue beyond 4 ns of production MD, and as such the Trp-cage SchNet free energy profile is not likely to be converged.

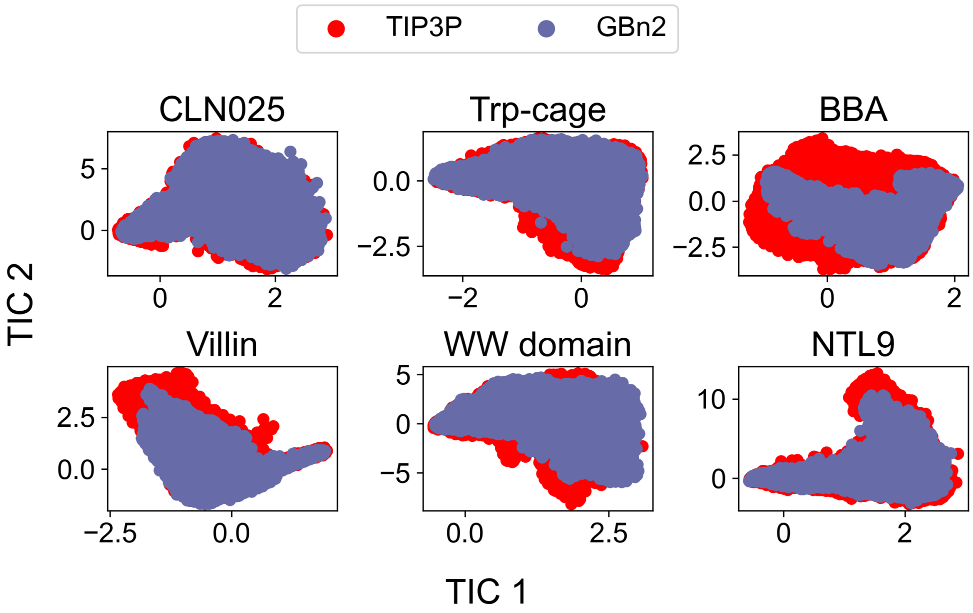

Figure S23: Explicit solvent (denoted as TIP3P) and GBn2 configuration TICs. TICA dimensionality reduction was performed according to the procedure detailed in section *Possible causes of lower accuracy for BBA, WW domain, & NTL9*. All TIP3P configurations for these 6 proteins were generated by Lindorff-Larsen et al.[S2] Noise configurations from the GBn2 umbrella sampling ensembles sufficiently cover the ranges sampled by the TIP3P data configurations for all proteins, except for BBA.

References

(S1) Jorgensen, W. L.; Chandrasekhar, J.; Madura, J. D.; Impey, R. W.; Klein, M. L. Comparison of simple potential functions for simulating liquid water. *J. Chem. Phys.* **1983**, *79*, 926–935.

(S2) Lindorff-Larsen, K.; Piana, S.; Dror, R. O.; Shaw, D. E. How Fast-Folding Proteins Fold. *Science* **2011**, *334*, 517–520.

(S3) Roe, D. R.; Cheatham, T. E. I. PTRAJ and CPPTRAJ: Software for Processing and Analysis of Molecular Dynamics Trajectory Data. *J. Chem. Theory Comput.* **2013**, *9*, 3084–3095.

(S4) Nguyen, H.; Roe, D. R.; Simmerling, C. Improved Generalized Born Solvent Model Parameters for Protein Simulations. *J. Chem. Theory Comput.* **2013**, *9*, 2020–2034.

(S5) Eastman, P.; Swails, J.; Chodera, J. D.; McGibbon, R. T.; Zhao, Y.; Beauchamp, K. A.; Wang, L.-P.; Simmonett, A. C.; Harrigan, M. P.; Stern, C. D. et al. OpenMM 7: Rapid development of high performance algorithms for molecular dynamics. *PLOS Comput. Biol.* **2017**, *13*, e1005659.

(S6) Best, R. B.; Zhu, X.; Shim, J.; Lopes, P. E. M.; Mittal, J.; Feig, M.; MacKerell, A. D. J. Optimization of the Additive CHARMM All-Atom Protein Force Field Targeting Improved Sampling of the Backbone ϕ, ψ and Side-Chain χ_1 and χ_2 Dihedral Angles. *J. Chem. Theory Comput.* **2012**, *8*, 3257–3273.

(S7) Huang, J.; Rauscher, S.; Nawrocki, G.; Ran, T.; Feig, M.; de Groot, B. L.; Grubmüller, H.; MacKerell, A. D. CHARMM36m: an improved force field for folded and intrinsically disordered proteins. *Nat. Methods* **2017**, *14*, 71–73.

(S8) Croitoru, A.; Park, S.-J.; Kumar, A.; Lee, J.; Im, W.; MacKerell, A. D.; Aleksan-

drov, A. Additive CHARMM36 Force Field for Nonstandard Amino Acids. *J. Chem. Theory Comput.* **2021**, *17*, 3554–3570.

(S9) Zhang, Z.; Liu, X.; Yan, K.; Tuckerman, M. E.; Liu, J. Unified Efficient Thermostat Scheme for the Canonical Ensemble with Holonomic or Isokinetic Constraints via Molecular Dynamics. *J. Phys. Chem. A* **2019**, *123*, 6056–6079.

(S10) Ding, X.; Zhang, B. Contrastive Learning of Coarse-Grained Force Fields. *J. Chem. Theory Comput.* **2022**, *18*, 6334–6344.

(S11) Maier, J. A.; Martinez, C.; Kasavajhala, K.; Wickstrom, L.; Hauser, K. E.; Simmerling, C. ff14SB: Improving the Accuracy of Protein Side Chain and Backbone Parameters from ff99SB. *J. Chem. Theory Comput.* **2015**, *11*, 3696–3713.

(S12) Ojaghlou, N.; Airas, J.; McRae, L. M.; Taylor, C. A.; Miller, B. R.; Parish, C. A. Understanding the Structure and Apo Dynamics of the Functionally Active JIP1 Fragment. *J. Chem. Inf. Model.* **2021**, *61*, 324–334.

(S13) Honda, S.; Yamasaki, K.; Sawada, Y.; Morii, H. 10 Residue Folded Peptide Designed by Segment Statistics. *Structure* **2004**, *12*, 1507–1518.

(S14) Maruyama, Y.; Koroku, S.; Imai, M.; Takeuchi, K.; Mitsutake, A. Mutation-induced change in chignolin stability from π-turn to α-turn. *RSC Adv.* **2020**, *10*, 22797–22808.

(S15) Brooks, B. R.; Brooks III, C. L.; Mackerell Jr., A. D.; Nilsson, L.; Petrella, R. J.; Roux, B.; Won, Y.; Archontis, G.; Bartels, C.; Boresch, S. et al. CHARMM: The biomolecular simulation program. *J. Comput. Chem.* **2009**, *30*, 1545–1614.

(S16) Jo, S.; Kim, T.; Iyer, V. G.; Im, W. CHARMM-GUI: A web-based graphical user interface for CHARMM. *J. Comput. Chem.* **2008**, *29*, 1859–1865.

(S17) Lee, J.; Cheng, X.; Swails, J. M.; Yeom, M. S.; Eastman, P. K.; Lemkul, J. A.; Wei, S.; Buckner, J.; Jeong, J. C.; Qi, Y. et al. CHARMM-GUI Input Generator for NAMD,

GROMACS, AMBER, OpenMM, and CHARMM/OpenMM Simulations Using the CHARMM36 Additive Force Field. *J. Chem. Theory Comput.* **2016**, *12*, 405–413.

(S18) Eastman, P. OpenMM PyTorch Plugin. 2023; https://github.com/openmm/openmm-torch, accessed on 2023-04-18.

(S19) Paszke, A.; Gross, S.; Massa, F.; Lerer, A.; Bradbury, J.; Chanan, G.; Killeen, T.; Lin, Z.; Gimelshein, N.; Antiga, L. et al. PyTorch: An Imperative Style, High-Performance Deep Learning Library. *arXiv* **2019**, arXiv:1912.01703, accessed on 2023-06-12.

(S20) Fey, M.; Lenssen, J. E. Fast Graph Representation Learning with PyTorch Geometric. *arXiv* **2019**, arXiv:1903.02428, accessed on 2023-06-12.

(S21) McGibbon, R. T.; Beauchamp, K. A.; Harrigan, M. P.; Klein, C.; Swails, J. M.; Hernández, C. X.; Schwantes, C. R.; Wang, L.-P.; Lane, T. J.; Pande, V. S. MDTraj: A Modern Open Library for the Analysis of Molecular Dynamics Trajectories. *Biophys. J.* **2015**, *109*, 1528–1532.

(S22) Schütt, K. T.; Sauceda, H. E.; Kindermans, P.-J.; Tkatchenko, A.; Müller, K.-R. SchNet – A deep learning architecture for molecules and materials. *J. Chem. Phys.* **2018**, *148*, 241722.

(S23) Kingma, D. P.; Ba, J. Adam: A Method for Stochastic Optimization. *arXiv* **2017**, arXiv:1412.6980, accessed on 2023-06-12.

(S24) Shirts, M. R.; Chodera, J. D. Statistically optimal analysis of samples from multiple equilibrium states. *J. Chem. Phys.* **2008**, *129*, 124105.

(S25) Tan, Z.; Gallicchio, E.; Lapelosa, M.; Levy, R. M. Theory of binless multi-state free energy estimation with applications to protein-ligand binding. *J. Chem. Phys.* **2012**, *136*, 144102.

(S26) Ding, X.; Vilseck, J. Z.; Brooks, C. L. A Fast Solver for Large Scale Multistate Bennett Acceptance Ratio Equations. *J. Chem. Theory Comput.* **2019**, *15*, 799–802.

(S27) Kunsch, H. R. The Jackknife and the Bootstrap for General Stationary Observations. *Ann. Stat.* **1989**, *17*, 1217–1241.

(S28) Virtanen, P.; Gommers, R.; Oliphant, T. E.; Haberland, M.; Reddy, T.; Cournapeau, D.; Burovski, E.; Peterson, P.; Weckesser, W.; Bright, J. et al. SciPy 1.0: fundamental algorithms for scientific computing in Python. *Nat. Methods* **2020**, *17*, 261–272.

(S29) Scott, D. W. *Multivariate density estimation: theory, practice, and visualization*; John Wiley & Sons, 2015.
